# The Yeast SR-Like Protein Npl3 Links Chromatin Modification to mRNA Processing

**DOI:** 10.1371/journal.pgen.1003101

**Published:** 2012-11-29

**Authors:** Erica A. Moehle, Colm J. Ryan, Nevan J. Krogan, Tracy L. Kress, Christine Guthrie

**Affiliations:** 1 Department of Biochemistry and Biophysics, University of California San Francisco, San Francisco, California, United States of America; 2 Department of Cellular and Molecular Pharmacology, California Institute for Quantitative Biomedical Research, University of California San Francisco, San Francisco, California, United States of America; 3 School of Computer Science and Informatics, University College Dublin, Dublin, Ireland; 4 J. David Gladstone Institutes, San Francisco, California, United States of America; 5 Department of Biology, The College of New Jersey, Ewing, New Jersey, United States of America; Harvard Medical School, United States of America

## Abstract

Eukaryotic gene expression involves tight coordination between transcription and pre–mRNA splicing; however, factors responsible for this coordination remain incompletely defined. Here, we explored the genetic, functional, and biochemical interactions of a likely coordinator, Npl3, an SR-like protein in *Saccharomyces cerevisiae* that we recently showed is required for efficient co-transcriptional recruitment of the splicing machinery. We surveyed the *NPL3* genetic interaction space and observed a significant enrichment for genes involved in histone modification and chromatin remodeling. Specifically, we found that Npl3 genetically interacts with both Bre1, which mono-ubiquitinates histone H2B as part of the RAD6 Complex, and Ubp8, the de-ubiquitinase of the SAGA Complex. In support of these genetic data, we show that Bre1 physically interacts with Npl3 in an RNA–independent manner. Furthermore, using a genome-wide splicing microarray, we found that the known splicing defect of a strain lacking Npl3 is exacerbated by deletion of *BRE1* or *UBP8*, a phenomenon phenocopied by a point mutation in H2B that abrogates ubiquitination. Intriguingly, even in the presence of wild-type *NPL3*, deletion of *BRE1* exhibits a mild splicing defect and elicits a growth defect in combination with deletions of early and late splicing factors. Taken together, our data reveal a connection between Npl3 and an extensive array of chromatin factors and describe an unanticipated functional link between histone H2B ubiquitination and pre–mRNA splicing.

## Author Summary

Pre-messenger RNA splicing is the process by which an intron is identified and removed from a transcript and the protein-coding exons are ligated together. It is carried out by the spliceosome, a large and dynamic molecular machine that catalyzes the splicing reaction. It is now apparent that most splicing occurs while the transcript is still engaged with RNA polymerase, implying that the biologically relevant splicing substrate is chromatin-associated. Here, we used a genetic approach to understand which factors participate in the coordination of transcription and splicing. Having recently shown that the Npl3 protein is involved in the recruitment of splicing factors to chromatin-associated transcripts, we performed a systematic screen for genetically interacting factors. Interestingly, we identified factors that influence the ubiquitin modification of histone H2B, a mark involved in transcription initiation and elongation. We show that disruption of the H2B ubiquitination/de-ubiquitination cycle results in defects in splicing, particularly in the absence of Npl3. Furthermore, the ubiquitin ligase, Bre1, shows genetic interactions with other, more canonical spliceosomal factors. Taken together with the myriad Npl3 interaction partners we found, our data suggest an extensive cross-talk between the spliceosome and chromatin.

## Introduction

Pre-mRNA splicing is a critical step in gene expression in which non-coding introns are removed from pre-mRNA and protein-coding exons are ligated together. This process is performed by the spliceosome, a dynamic ribonucleoprotein particle that, in yeast, consists of 5 snRNAs and over 80 proteins that cooperate to recognize and splice target mRNAs [1]. Recent evidence reveals that mRNA splicing *in vivo* is largely co-transcriptional, and occurs while elongating RNA polymerase II (PolII) is still associated with chromatin [2]–[4]. The basic unit of chromatin is 146 base pairs of DNA wound around a histone octamer to form a nucleosome, arrays of which can be further compacted to form higher-order chromatin structure. A plethora of chromatin remodeling and histone modifying machines are now known to be integral parts of the gene expression process [5]. While much has been learned about the molecular mechanisms of pre-mRNA splicing from *in vitro* systems [6], a full understanding of the regulation of spliceosome assembly and catalysis will require an appreciation of the complex landscape of the chromatinized template, along which splicing occurs.

To approach this question, we built upon our recent observation that the SR-like protein Npl3 promotes efficient splicing of a large subset of genes via co-transcriptional recruitment of U1 and U2 snRNPs [7]. SR and hnRNP proteins in metazoa are best understood for their role in alternative and constitutive splicing, although they have also been implicated in additional steps in gene expression, including mRNA export, translation, and even transcription itself [8]–[10]. Despite the fact that there are few examples of alternative splicing in *S. cerevisiae*, this yeast contains three genes with a canonical SR protein domain structure: one or more RNA recognition motifs and a domain enriched in arginine-serine dipeptides [9], [11]. We recently demonstrated that deletion of *NPL3* specifically, but not the others, impacts splicing; interestingly, the affected genes are almost exclusively those encoding ribosomal proteins, and make up the largest class of intron-containing genes in budding yeast [7]. Npl3 appears to be appropriately poised to coordinate events in gene expression: it is recruited to chromatin early during transcription [12] stimulates transcription elongation [13]–[16] co-purifies with elongating PolII [12], [14] via its interaction with the C-terminal domain [14], and remains associated with mRNA after processing is completed [17], [18].

Here, in order to understand how Npl3 might choreograph gene expression events in *S. cerevisiae*, we systematically analyzed genetic interactions of a strain lacking Npl3. We uncovered genetic interactions between the *npl3*Δ allele and genes involved in transcription and chromatin modification, including factors involved in histone H2B ubiquitination: the E3 ubiquitin ligase, Bre1 [19], [20], and corresponding ubiquitin protease, Ubp8 [21]–[23]. In addition, we show that Npl3 physically interacts with Bre1. Splicing-sensitive microarray experiments reveal that disabling the H2B ubiquitination pathway by deletion of *BRE1* or *UBP8*, or point mutation of H2B, exacerbates the known splicing defect of an *npl3*Δ strain. Furthermore, we observed an Npl3-independent connection between Bre1 and splicing, as deletion of *BRE1* impairs the splicing of a subset of pre-mRNAs and, in combination with deletions of individual splicing factors, causes severe synthetic growth defects. Thus, our data functionally link H2B ubiquitination by Bre1 to pre-mRNA splicing and more broadly suggest that the coordination of transcription and splicing may be aided by crosstalk between Npl3 and chromatin metabolism.

## Results

### A genetic link between *NPL3* and chromatin modification

**Figure 1:**
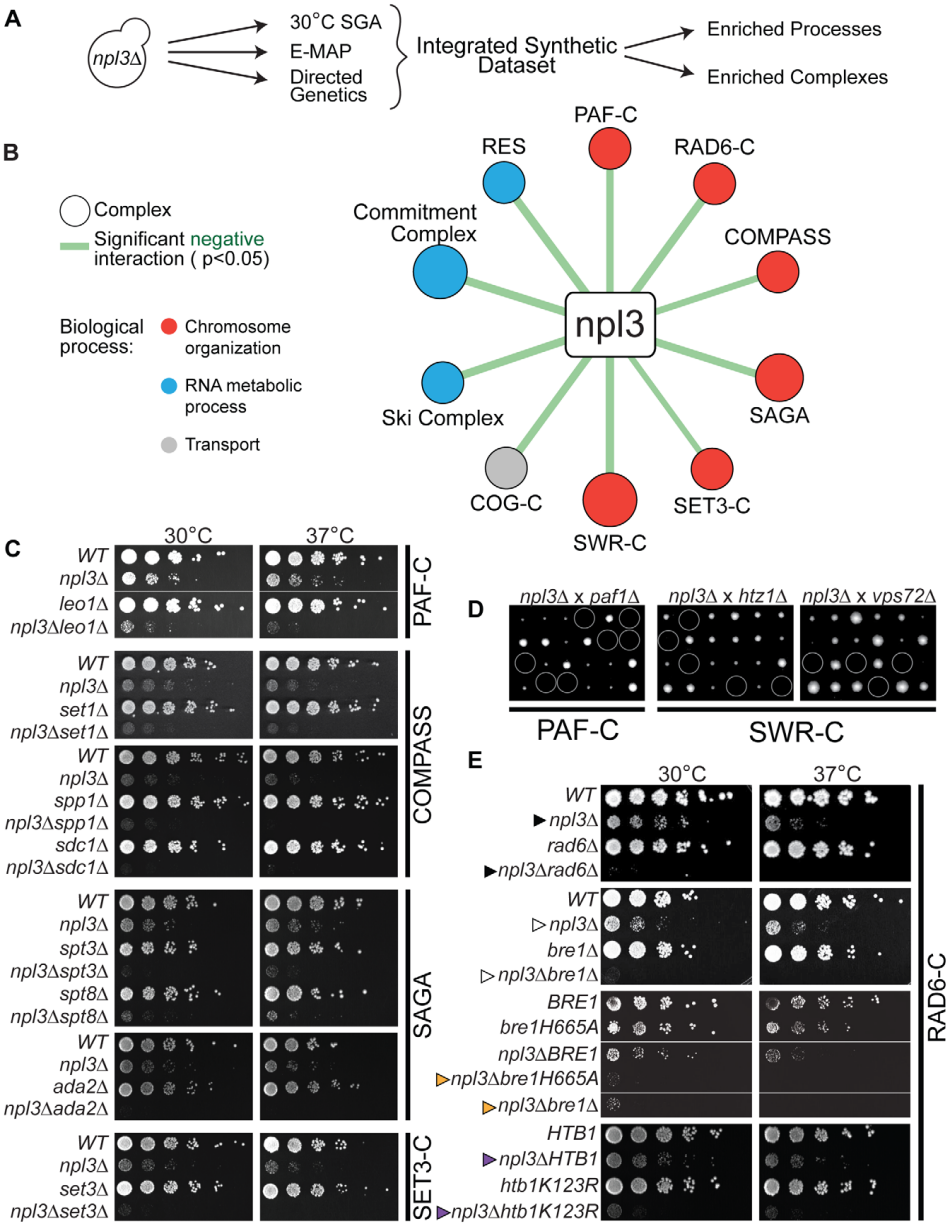
Extensive negative genetic interactions with *npl3* Δ connect *NPL3* to chromatin biology. (A) Work flow for analysis of the integrated synthetic dataset. Synthetic genetic array technology was used to screen ∼4800 non-essential genes whose deletion conferred lethality to *npl3*Δ at 30°C. These results were augmented by including genes exhibiting a genetic interaction score of ≤−2.5 with *npl3*Δ [26] and genes identified as synthetic sick or lethal using tetrad dissection and serial dilution (directed genetics). (B) Statistically significant negative interactions between *NPL3* and known complexes. Statistical analysis identified the indicated complexes as having subunits significantly enriched (P<0.05) in the integrated synthetic dataset. Size of circle is based on number of subunits whose deletion exacerbates the growth defect of *npl3*Δ and thickness of line scales with the significance of the enrichment. Circles are color-coded based on the biological process to which the complex belongs. (C) Synthetic growth analyses with *npl3*Δ and genes implicated in chromatin biology. Each panel shows a double mutant strain, cognate single deletions strains and a corresponding wild-type that have been serially diluted onto rich medium and grown at the indicated temperatures. Double mutants were isolated after tetrad dissection. To the right of panels is the name of the complex to which the single chromatin mutants belong. (D) Tetrad dissection analyses with *npl3*Δ. Tetrad dissection plates from the indicated crosses are shown with the inviable spore circled. Replica plating to infer genotype later showed that inviable spores are the double mutants. (E) Synthetic growth analyses with *npl3*Δ and genes encoding the H2B ubiquitination machinery. Shown are serial dilutions of the indicated strains after incubation at the indicated temperatures. Genotypes not originally tested in the SGA are *htb1K123R* and *bre1H665A*. The *BRE1* and *bre1H665A* strains contain a *bre1* deletion covered by a plasmid encoding the indicated *bre1* allele. Arrowheads refer to comparisons made in the text.

The SR-like protein Npl3 has multiple roles in the regulation of gene expression, including in pre-mRNA splicing, 3′ end processing, and mRNA export. To further interrogate this multifunctional factor, we used synthetic genetic array (SGA) technology [24], [25] to screen ∼4,800 non-essential yeast genes for those whose deletion conferred synthetic lethality (SL) or very synthetic sick (SS) growth phenotypes in an *npl3*Δ strain. Since an *npl3*Δ strain grows more slowly than wild-type at 30°C, and this defect is exacerbated at 37°C (*e.g.*, see Figure 1C, top panels), we performed the screen at both temperatures to maximize coverage. The analysis revealed strong negative interactions between *NPL3* and 83 (1.7% of total) and 333 (6.9% of total) genes after growth at 30°C and 37°C, respectively (see Table S1).

To validate a subset of genetic interactions identified by this high-throughput approach, we generated the cognate double mutant strains using tetrad dissection. In order to refine our list of genetically interacting factors, we included additional subunits from complexes represented in the results of the screen. A list of the most stringent synthetic interaction partners (identified in the 30°C SGA and directed genetics) was integrated with those from a previously published quantitative RNA processing Epistatic Mini Array Profile (E-MAP) [26] to generate a more comprehensive set of *NPL3* SS/SL genetic interactions (Figure 1A and Materials and Methods). These negative genetic interactions were highly enriched for genes that function in RNA metabolism (Table S2), consistent with what was previously known about Npl3 function in mRNA processing [7], [12], [13], [16]–[18], [27]–[31]. In addition, there was an enrichment of genetic interactions with genes implicated in “chromosome organization” and “transcription,” including components of the chromatin remodeling SWR Complex [32]–[34], the transcriptional elongation PAF Complex [35]–[38], and multiple histone modification complexes, including COMPASS [39], SAGA [40], and the SET3 Complex [41] (Figure 1B, 1C and 1D and Table S3). We note that of these, the SWR1 and SAGA Complexes have previously been implicated in pre-mRNA splicing [42], [43], highlighting the ability of the Npl3 screen to identify factors involved in chromatin-splicing crosstalk.

The screens also showed that deletion of either *RAD6* (Figure 1E *cf.* closed triangles) or *BRE1* (Figure 1E *cf.* open triangles) led to synthetic sickness/lethality in an *NPL3* deletion strain. These factors catalyze the mono-ubiquitination of lysine 123 on histone H2B; specifically, Bre1 is the E3 ubiquitin ligase and Rad6 is its corresponding E2 ubiquitin-conjugating enzyme [19], [20], [44], [45]. We found that inactivating Bre1 ubiquitin ligase activity via a point mutation in its RING domain (*bre1H665A*) [19] exacerbated the growth defect of an *npl3*Δ strain to the same extent as a full deletion of *BRE1* (Figure 1E, *cf.* orange triangles), suggesting that the genetic interaction is connected to the ligase activity of Bre1. Many nuclear enzymes act not only on histones but on other substrates as well, and, in fact, histone H2B is not the only ubiquitination target of Bre1 [46]. To ask whether the Npl3-Bre1 genetic interaction is due to the loss of H2B ubiquitination specifically, we tested whether a mutation of the target residue in H2B would phenocopy a deletion of *BRE1*. Indeed, the *htb1K123R* point mutant also profoundly exacerbated the growth defect of *npl3*Δ (Figure 1E *cf.* purple triangles). Taken together, these data provide strong evidence that H2B ubiquitination can account for the genetic interaction of the RAD6 Complex with *NPL3*.

The PAF Complex and COMPASS have previously been shown to function in the same histone modification pathway as the Bre1 [47]–[50]. The PAF Complex is required for H2B ubiquitination [49], [50]; thus, the synthetic lethality we observed between *NPL3* and components of the PAF Complex (Figure 1C and 1D) was consistent with the genetic interactions we observed with the Bre1. H2B ubiquitination is, in turn, required for trimethylation of histone H3 lysine 4 (H3K4) by COMPASS [39], [51]–[53] and lysine 79 (H3K79) by Dot1 [54]–[57]. However, we found no genetic interaction between *NPL3* and point mutations of H3K4 or H3K79 (data not shown), suggesting that loss of these chromatin marks is unlikely to underlie the synthetic sickness in the *npl3*Δ*bre1*Δ double mutant.

**Figure 2:**
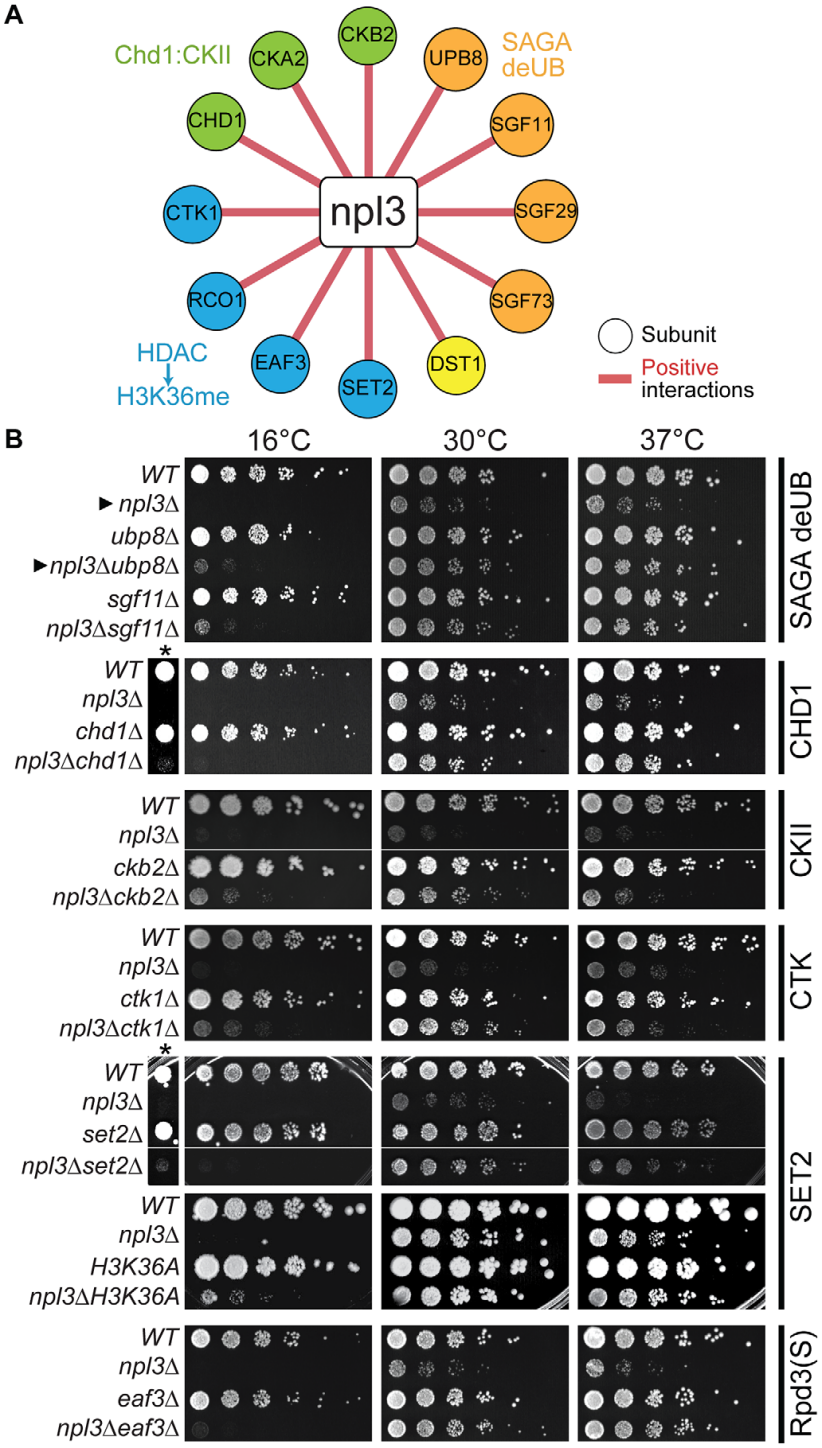
The *npl3*Δ growth defect is suppressed by mutations in genes encoding transcription and chromatin factors. (A) A subset of suppressive *npl3*Δ genetic interactions relevant to chromatin biology identified as allowing growth to *npl3*Δ in the SGA performed at 16°C. Genes are arranged by complex or pathway. Full list is available in Table S4. (B) Suppressive growth analyses with *npl3*Δ and genes implicated in chromatin biology. Shown are serial dilutions of the indicated strains grown at the indicated temperatures. All double mutants were generated by tetrad dissection. *H3K36A* was not originally tested in the SGA. Asterisk marks a higher-contrast image to better visualize suppression at 16°C. Arrowheads refer to comparisons made in the text.

Given that maintaining H2B ubiquitination is critical in the absence of *NPL3*, it follows that mutations in genes required for the removal of this chromatin mark might suppress the *npl3*Δ growth defect. To investigate this in an unbiased fashion, we made use of the fact that *NPL3* deletion causes lethality when yeast are grown at 16°C (e.g., see Figure 2B, top panel); this allowed us to screen for mutants that restore growth to an *npl3*Δ strain at 16°C. This screen identified 105 (2.1% of total) and 699 (14.4% of total) suppressors after 4 and 8 days of growth, respectively (Table S4), and a number of these suppressors have previously been implicated in transcription and chromatin modification (Figure 2A). We then generated a number of the double mutants using tetrad dissection and validated the suppressive genetic interactions using serial dilution (Figure 2B). In agreement with our expectation, the data from this screen showed that deletion of *UBP8*, which encodes an H2B de-ubiquitinase [21]–[23], restored viability to a strain lacking Npl3 (Figure 2B *cf.* closed triangles). In further support of these observations, the SGA also identified *SGF11* and *SGF73* as genes whose deletion suppresses *npl3*Δ; these factors are part of a module of the SAGA Complex with Upb8, and are also implicated in gene activation by H2B de-ubiquitination [21], [58]–[61]. Taken together, this dataset shows that the *npl3*Δ strain is particularly sensitive to deletion of genes affecting the H2B ubiquitination pathway (Figure 1 and Figure 2) and opens the possibility that H2B ubiquitination is important for an Npl3-dependent process.

Interestingly, deletions of genes in other modules of SAGA required for either histone acetylation (Ada2 and Gcn5) or for association of the SAGA complex with promoters (*i.e.,* the TBP regulatory module, Spt3 and Spt8; reviewed in [62]) exacerbated, rather than suppressed, the *npl3*Δ growth defect (Figure 1C and Table S1). The divergent genetic interactions confirm the functionally separable nature of the SAGA sub-modules [58] and highlight that a connection exists between Npl3 and H2B mono-ubiquitination that is functionally distinct from other chromatin marks.

### Npl3 physically interacts with Bre1

**Figure 3:**
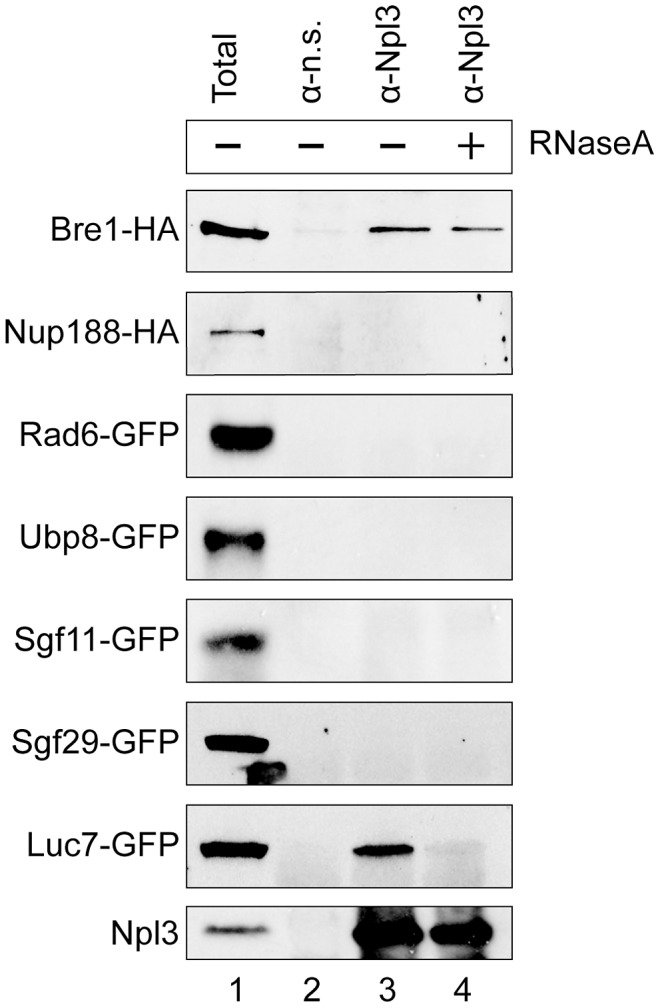
Npl3 physically interacts with Bre1. Co-immunoprecipitation analyses of Npl3 and members of the histone H2B ubiquitination machinery. Whole cell extracts from strains with the indicated proteins endogenously tagged with HA or GFP were immunoprecipitated with an α-Npl3 antibody [63] or non-specific antibody (α–n.s.). Western blot using α-HA or α-GFP from each co-IP experiment is shown. The sensitivity of the interaction to RNase (lane 4, +RNaseA) was determined by treating lysates with RNase A prior to immunoprecipitation. Lane 1 shows 1/60 total sample for each lysate. Bottom panel confirms presence of Npl3 in the immunoprecipitate.

Given the robust genetic interactions we observed between *NPL3* and genes involved in H2B ubiquitination, we performed co-immunoprecipitation assays of the corresponding proteins to test if they physically interact. We had previously shown that Npl3 co-immunoprecipitated components of the U1 snRNP [7]. Here, we immunoprecipitated endogenous Npl3 from whole-cell extract using a polyclonal antibody directed against Npl3 [63] and then probed the precipitate for endogenously tagged forms of Bre1, Ubp8, and Sgf11 as well as positive and negative controls (a U1 protein, Luc7, and Nup188, respectively). Although there is precedent for some interaction specificity with the E3 Bre1 over the E2 Rad6 [46], we also tested for an interaction with Rad6. As shown in Figure 3, only Bre1 and Luc7 but not Nup188, Rad6, Ubp8 or Sgf11, co-immunoprecipitated with Npl3.

It is known that Npl3 is an RNA-binding protein, and its interaction with some components of the splicing machinery is RNA-dependent [7]. To test whether the observed interaction with Bre1 is mediated by RNA, we treated the extracts with RNaseA prior to the immunoprecipitation. We consistently found that a population of Bre1 interacts with Npl3 in an RNase-independent manner (Figure 3 *cf.* lanes 3 and 4, top panel). These data indicate that Npl3 can physically interact with Bre1, consistent with previous data from high-throughput proteomic analyses [64].

### Deletion of *BRE1* or *UBP8* exacerbates the *npl3*Δ splicing defect

The genetic data connecting *NPL3* and the H2B ubiquitination machinery lend support for two possible models. One model predicts that Npl3 will affect H2B ubiquitination; we therefore measured the global percentage of ubiquitinated H2B but found the *npl3*Δ strain indistinguishable from wild-type (Figure S1). An alternative interpretation of the genetic data is that the H2B ubiquitination cycle is important for an Npl3-dependent process. We previously reported [7] that a strain lacking Npl3 accumulates a subset of pre-mRNAs, consisting primarily of the ribosomal protein genes (RPGs), whose splicing efficiency might be expected to affect growth rate. Given that deletion of *BRE1* exacerbates the *npl3*Δ growth defect, we tested whether deleting *BRE1* exacerbates the *npl3*Δ splicing defect.

**Figure 4:**
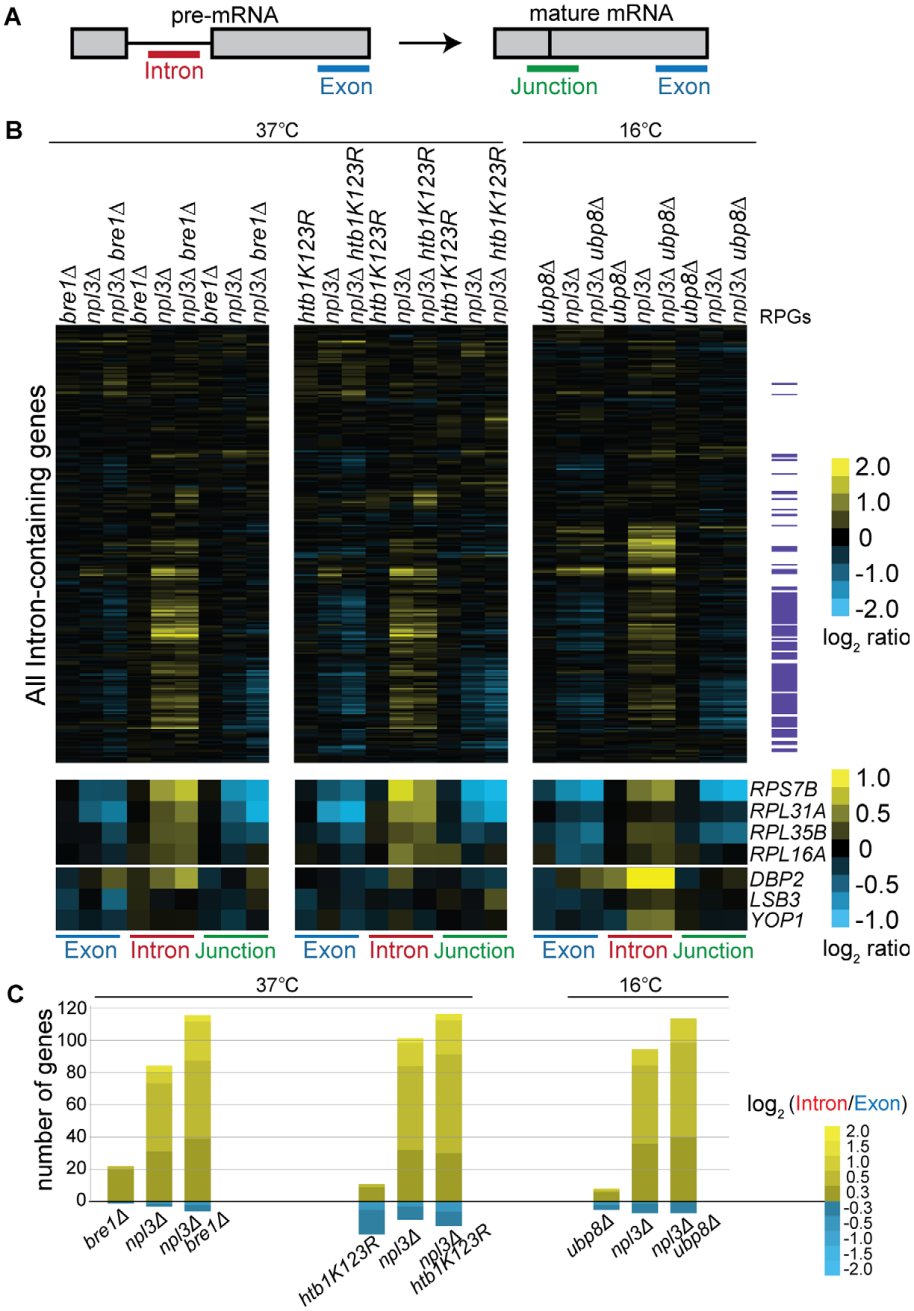
Splicing is sensitive to Npl3 and the H2B ubiquitination cycle. (A) Schematic of probes contained on the splicing microarray. (B) Splicing profile of single or double mutant strains compared to wild-type. Cultures of the indicated strains and isogenic wild-type strains were grown to mid-log phase at 30°C and shifted to the indicated temperature; cDNA from single and double mutant strains were competitively hybridized on the microarray against that from an isogenic wild-type. The heat map shows the log_2_-ratio for each gene feature of the indicated strain compared to wild-type. Gene order along the y-axis is the same for all arrays. Transcripts that encode the ribosomal protein genes (RPGs) are highlighted in purple to the right of the heat maps. Data for example genes are replicated below the genome-wide heat map to show splicing defects and exacerbation of defects at individual RPGs and non-RPGs. (C) Histogram of log_2_-based Intron Accumulation Index scores. An Intron Accumulation Index value was calculated for each intron-containing gene by normalizing the intron change to exon change (see Materials and Methods). Histogram shows the number of genes with an Intron Accumulation Index score greater than 0.3. Heat map within histogram bars shows distribution of the severity of splicing defect.

We used our splicing-sensitive microarray platform [65], which contains oligos that hybridize to the terminal exon, the intron, and the exon-exon junction of each intron-containing gene, in order to detect total mRNA, pre-mRNA, and mature mRNA, respectively (Figure 4A). For each genotype, the heat map (Figure 4B) reports fold changes in signal intensity of these three RNA species for each intron-containing gene as compared to a wild-type strain. As expected, our experiments showed that a strain lacking Npl3 accumulated RPG pre-mRNAs (Figure 4B *npl3*Δ, see yellow in Intron feature; RPGs highlighted in purple on right). Notably, the pre-mRNA accumulation in the *npl3*Δ strain was increased at many RPGs when *BRE1* was also deleted (Figure 4B *cf. npl3*Δ and *npl3*Δ*bre1*Δ, Intron feature), suggesting that Bre1 is important for the splicing of many Npl3-dependent genes. We note that this effect is complex, and is accompanied by changes in total mRNA (Figure 4B *cf. npl3*Δ and *npl3*Δ*bre1*Δ, Exon feature). Because both Npl3 and Bre1 have been shown to have effects on transcription itself [13]–[16], [66]–[68], we normalized for changes in exon level by calculating an Intron Accumulation Index [69] (see Materials and Methods) for each intron-containing gene (Figure S2 and Table S5). The histogram of genes with an Intron Accumulation Index of greater than 0.3 (Figure 4C), shows that even when normalized for changes in transcript levels, the total number of genes with a splicing defect, as well as the severity of the defect, is increased in the *npl3*Δ*bre1*Δ strain as compared to *npl3*Δ alone.

We also found that in the presence of wild-type Npl3, a strain lacking *BRE1* has a mild but reproducible splicing defect (Figure 4B, *bre1*Δ –shown is an average of 5 biological replicates). While the majority of pre-mRNAs are not affected by the deletion of *BRE1*, a small subset of pre-mRNAs accumulates in *bre1*Δ at 37°C (Figure 4B, *e.g.*, *DBP2, LSB3, YOP1*). This suggests that Bre1 has a role in pre-mRNA splicing, independent of the sensitivity caused when *NPL3* is deleted. This finding was confirmed when we calculated Intron Accumulation Indices for a strain lacking *BRE1:* a small number of genes exhibit defective splicing in the *bre1*Δ strain (Figure 4C and Figure S2). We validated these splicing defects for several genes using a qPCR assay (Figure S3). The lack of a significant growth defect in the *bre1*Δ strain (Figure 1E) is consistent with the idea that yeast can tolerate a modest splicing defect at a small number of non-RPGs.

If the splicing defect exacerbation we observed with *npl3*Δ*bre1*Δ was due to loss of H2B ubiquitination, we would then expect this exacerbation to be phenocopied by a strain with the H2B lysine to arginine point mutant used earlier (Figure 1E). We did, in fact, find that the *htb1K123R* point mutation exacerbated the splicing defect observed in the *npl3*Δ mutant at many genes (Figure 4B), further implicating the ubiquitination of H2B in splicing. This is also evident when normalizing for the changes in exon levels in the *npl3*Δ*htb1K123R* strain (Figure 4C). In plotting the Intron Accumulation Index values of this strain, we find that the subset of affected genes overlaps extensively with the subset of genes affected in the *npl3*Δ*bre1*Δ double mutant (Figure S2).

We have shown that deletion of *UBP8* partially suppresses the *npl3*Δ growth defect, and this is most pronounced at 16°C (Figure 2B). We therefore tested whether deleting *UBP8* would suppress the splicing defect of a strain lacking Npl3, as predicted by the genetic interaction. Surprisingly, deletion of *UBP8* instead exacerbated the splicing defect observed in the *npl3*Δ strain (Figure 4B *cf. npl3*Δ and *npl3*Δ*ubp8*Δ), implying that the growth suppression is related to some other function of Npl3. Notably, however, these microarray results indicate that in the absence of Npl3, the complete cycle of H2B ubiquitination and de-ubiquitination is required for efficient splicing.

To begin to investigate how Bre1 affects splicing, we used chromatin immunoprecipitation (ChIP) to test the prediction that Bre1 is required for association of the splicing machinery. However, we did not observe a significant Bre1-dependent decrease in U1 (Prp42), Mud2, or U2 (Lea1) association with genes whose splicing was inhibited in *bre1*Δ or *npl3*Δ*bre1*Δ strains (data not shown), suggesting an alternative mechanism by which Bre1 modulates splicing (see discussion).

### Synthetic sickness between *BRE1* and early and late splicing factors

**Figure 5:**
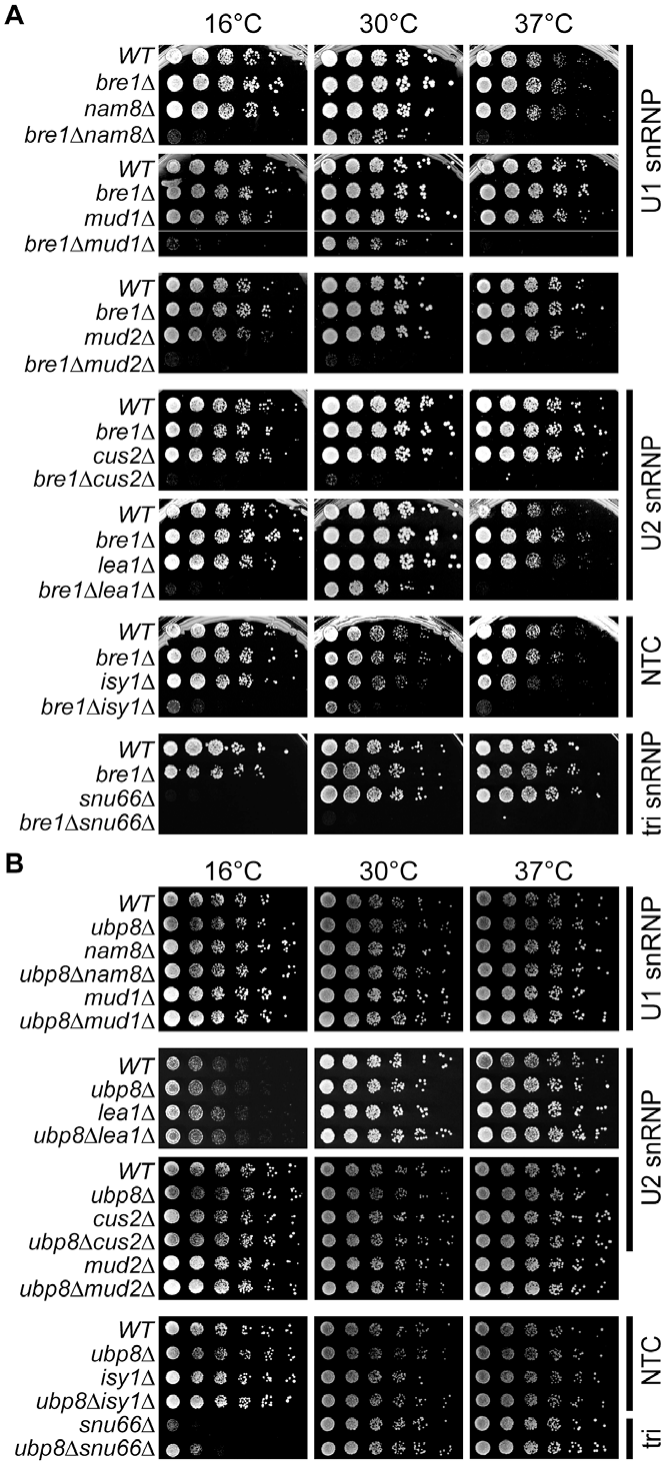
Genetic interactions between H2B ubiquitination machinery and canonical splicing factors. (A) Synthetic growth analyses between *bre1Δ* and genes encoding splicing factors. Double mutants were generated by tetrad dissection; log-phase cultures of the indicated strains were serially diluted and grown at the indicated temperatures. To the right of panels is the name of the spliceosomal complex to which the splicing factor mutants belong. NTC: Nineteen Complex. (B) Growth analyses of *ubp8Δ* and genes encoding splicing factors. Double mutants were generated and analyzed as in (A).

In light of our data showing that a *bre1*Δ strain exhibited a mild splicing defect, we carried out directed genetic analyses to test for interactions between *BRE1* and genes encoding other splicing factors, particularly those that genetically interact with Npl3 [7]. Just like a deletion of *NPL3*, deleting *BRE1* caused synthetic sickness when combined with deletion of *NAM8* (U1 snRNP), *MUD2*, *LEA1* (U2), or *SNU66* (U5), further connecting Bre1 functionally with splicing (Figure 5A). Interestingly, the growth of the *bre1*Δ strain was also compromised by deletion of the U2 snRNP component *CUS2*, which does not genetically interact with *npl3*Δ [7]. Thus, although we approached these experiments through the lens of Npl3, these genetic observations provide further support that Bre1 has independent interactions with the splicing machinery. Consistent with a lack of splicing defect upon *UPB8* deletion, we and others generally did not observe genetic interactions between *UBP8* and early or late splicing factors (Figure 5B and [70]). There is one notable exception however; deletion of *UBP8* suppressed the *snu66*Δ cold-sensitive growth defect (Figure 5B). Taken together, these data highlight the fact that the H2B ubiquitination pathway is linked to splicing, even in the presence of wild-type Npl3.

## Discussion

While the textbook view of gene expression presents transcription, pre-mRNA processing, export, and translation as independent events, they appear to be closely coordinated in the cell. Discerning the mechanism of this coordination within the broader program of gene expression presents a daunting experimental challenge. In multicellular eukaryotes, SR and hnRNP proteins are thought to regulate gene expression, in part, by integrating mRNA biogenesis steps [9], [10], [71]–[73]. In budding yeast, Npl3 has numerous roles, but is the only such protein that affects splicing [7]. Our lab and others have shown that Npl3 facilitates the co-transcriptional recruitment of early splicing factors to nascent transcripts [7] and itself associates with elongating polymerase [12], [14]. Therefore, we approached this complex problem by conducting a systematic screen of non-essential genes to define the interacting partners of this potential coupling factor.

**Figure 6:**
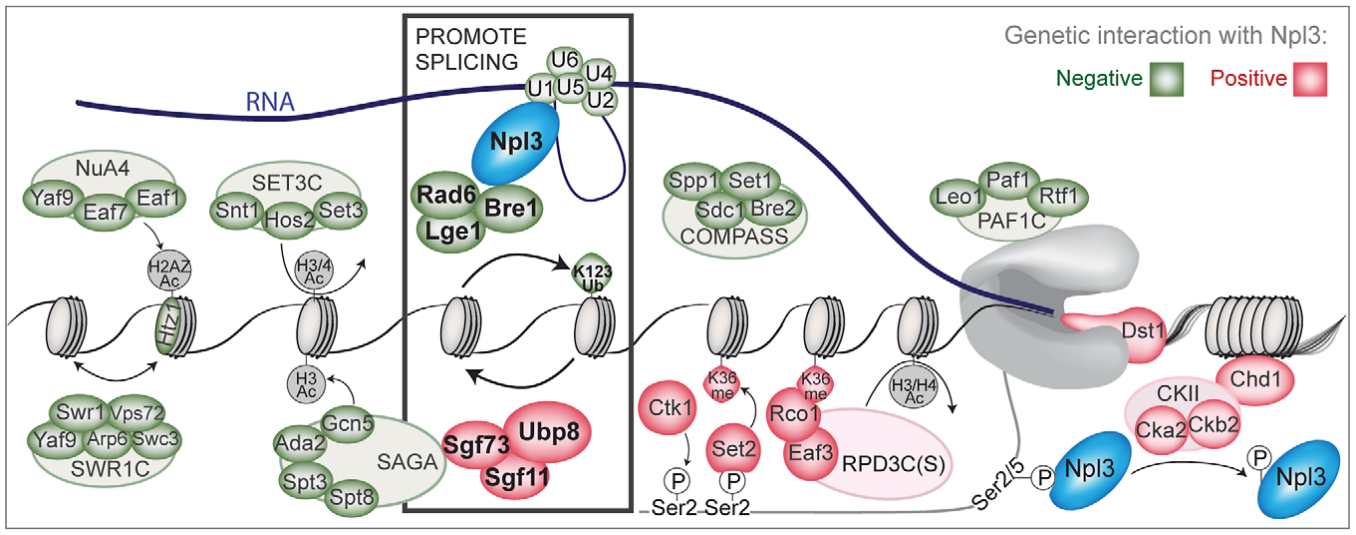
A chromatin-centered survey of Npl3 genetic interactions. Summary of chromatin and transcription factors that exhibit genetic interactions with a deletion of *NPL3*. Colored ovals represent subunits identified in the SGA screens or by directed genetics; red indicates a suppressive (positive) interaction and green indicates a synthetic (negative) interaction. Outlined ovals refer to the complex that individual subunits belong to. K123Ub refers to the *htb1K123R* point mutant. K36me refers the *hht1K36A* point mutant. Grey or white indicates the genetic interaction was not tested. Physical interactions tested by co-IP are Npl3:Bre1 (Figure 3) and Npl3:U1 [7]. Bold rectangle indicates factors shown in this paper and [7] to promote splicing; whether the presence of Npl3 can influence local H2B ubiquitination levels or dynamics remains unresolved. The PolII C-terminal domain is drawn in grey.

We found that Npl3 genetically interacts with a number of genes implicated in chromatin metabolism and transcription. We further characterized one set of interacting factors, namely those involved in histone H2B ubiquitination, in what is, to our knowledge, the first set of genome-wide splicing experiments on histone modifier mutants in *S. cerevisiae*. Using splicing-sensitive microarrays, we showed that Npl3 links the H2B ubiquitination cycle to the splicing efficiency of many transcripts. The connection between H2B ubiquitination and splicing also exists independently of Npl3, as a strain lacking *BRE1* exhibits both a mild splicing defect and genetic interactions with deletions of genes encoding early and late splicing factors. Finally, the full complement of genetic interactions we describe (Figure 6) provides multiple entry points for future investigation into the coupling of chromatin modification and mRNA processing.

### An Npl3-dependent role for the histone H2B ubiquitination cycle in pre–mRNA splicing

Our genetic screens revealed that a number of Npl3 genetic interactions center on the histone H2B ubiquitination cycle. Specifically, mutant strains that lack wild-type levels of ubiquitinated H2B (*rad6*Δ, *bre1*Δ, *lge1*Δ, *htb1K123R*, *paf1*Δ, *cdc73*Δ, and *leo1*Δ) exacerbate the growth defect of an *npl3*Δ strain at all temperatures tested (Figure 1). We also observed a physical interaction between Npl3 and Bre1 by co-immunoprecipitation (Figure 3) and showed that the splicing defect caused by deletion of *NPL3* is exacerbated by the additional deletion of *BRE1* or mutation of H2B (*htb1K123R*), thus implicating H2B lysine 123 mono-ubiquitination in splicing (Figure 4). We previously demonstrated that Npl3 primarily affects the splicing of RPGs [7]; here, we see that in the sensitized background of a strain in which RPG splicing is made limiting (*npl3*Δ), the histone H2B ubiquitination cycle is an important contributor to RPG splicing.

Recent studies have shown that deletion of components of the cap-binding complex (CBC) or commitment complex causes defective splicing of the *SUS1* pre-mRNA [70], [74]. Sus1 is a recently discovered component of the histone de-ubiquitination module of SAGA [75] and if the *SUS1* transcript is not properly spliced, it leads to elevated levels of ubiquitinated H2B. Given the physical [29], [76] and genetic (Table S1 and [76]) connections between Npl3 and the CBC, we sought to determine whether Npl3 also affects *SUS1* splicing and, therefore, H2B ubiquitination. However, Hossain, *et al.* have recently shown that deletion of *NPL3* has no effect on *SUS1* splicing [74], a result we independently confirmed in our *npl3*Δ strain (Figure S4). Furthermore, we extended this analysis and determined that, unlike in *cbc*Δ strains, global levels of ubiquitinated histone H2B are not discernibly altered in the *npl3*Δ strain (Figure S1). While we cannot rule out a change in the dynamics of the ubiquitination cycle or gene-specific effects, our microarray results support a model in which the full histone ubiquitination cycle promotes RPG splicing, a process that becomes critical in the absence of *NPL3*. Along these lines, it is noteworthy that data from Schulze *et al.* and Shieh *et al.* have revealed that chromatin over these genes is enriched for ubiquitinated H2B [77], [78].

We also identified suppressive genetic interactions between *NPL3* and genes responsible for removal of ubiquitin from H2B (*ubp8*Δ, *sgf11*Δ, and *sgf73*Δ), suggesting that H2B de-ubiquitination is also linked to Npl3 function. Surprisingly, however, deletion of *UBP8* did not suppress the splicing defect in *npl3*Δ, but rather exacerbated it (Figure 4). Thus, it seems the positive genetic interaction may be due to Ubp8 involvement in a splicing-independent function of Npl3.

The exacerbation seen in the microarray experiments shows that both halves of the cycle of H2B ubiquitination and de-ubiquitination are required for optimal splicing, as is the case for transcriptional activation [23]. Likewise, both halves of the H3 acetylation and deacetylation cycle, performed by Gcn5 and Hos2/3, respectively, promote spliceosome assembly at the *ECM33* gene [43], [79]. Thus, these two examples point to a general function of dynamic histone modification cycles in maintaining fine control over co-transcriptional splicing, and may explain the synthetic lethality we observed between *NPL3* and the acetylation module of the SAGA Complex (Figure 1C and Table S1).

### An Npl3-independent role for Bre1 in pre–mRNA splicing

We found that even in the presence of wild-type *NPL3*, Bre1 has genetic connections to the splicing machinery as a whole. Specifically, we found that deletion of *BRE1* causes growth defects in early and late splicing factor deletion backgrounds (particularly at extreme temperatures; Figure 5, 16°C and 37°C), which alone show little to no growth defect. These negative genetic interactions can indicate two alternative but not mutually exclusive models for a functional relationship between the H2B ubiquitination and splicing machineries. One model is based on the fact that deletion of specific splicing factors is known to increase the levels of ubiquitinated H2B [74], a phenotype that should be relieved by deletion of *BRE1*, the sole H2B ubiquitin ligase [19], [20]. Because this model predicts an epistatic or positive genetic interaction between *BRE1* and the genes that encode splicing factors, the negative genetic interactions that we actually observe (Figure 5 and [70]) require an alternative model, perhaps one in which the growth defects are due to poorer overall splicing efficiency in these strains. Indeed, deletion of *BRE1* alone caused a modest but reproducible splicing defect, seen in the microarray in Figure 4. A large fraction of Bre1-dependent splicing events involve non-RPGs, and thus define a distinct role for Bre1 in splicing, apart from Npl3. Shieh *et al.* [78] recently found that the pattern of this modified histone at non-RPGs shows a remarkable demarcation of intron/exon structure: low levels in the intron, followed by a marked increase at the intron – exon boundary. While the functional significance of this pattern of H2B ubiquitination is unknown, we propose that it may be relevant for the splicing of non-RPGs, as gauged by the splicing defect in a strain that no longer has this mark.

We note that the single mutant *htb1K123R* has a milder splicing defect than the *bre1*Δ strain (Figure 4C and Figure S2), opening the possibility of an additional role of Bre1 in splicing that is independent of H2B ubiquitination. Indeed, Bre1-dependent ubiquitination of Swd2, a protein in both COMPASS and the Cleavage and Polyadenylation Stimulatory Factor complex [46], has been shown to regulate mRNA export from the nucleus [80]. Npl3 has previously been implicated in mRNA export [17], [18], [27], [28] in a strain background where Npl3 is an essential protein. However, our data argue against the possibility that the genetic interactions we observed here are due to an adverse effect on mRNA export. In the present strain background (S288C), in which Npl3 is non-essential, the *npl3*Δ strain does not exhibit the nuclear localization of bulk poly-adenylated mRNA characteristic of an export defect (Figure S5); nor does further deletion of *BRE1* in an *npl3*Δ strain cause an export defect (Figure S5). Furthermore, we found that the *npl3S411A* phosphorylation mutation, which blocks 3′ end formation [13], [14] and mRNA export [17], does not cause a block in pre-mRNA splicing (Figure S6). This argues against the reported splicing defects being the indirect result of feedback from these downstream defects in mRNA processing.

We tested the prediction that Bre1, like Npl3, promotes spliceosome recruitment, but found that deletion of *BRE1* did not affect the association of U1 (Prp42), Mud2, or U2 (Lea1) with chromatin at genes whose splicing is dependent on Bre1 (data not shown). It may be that H2B ubiquitination is required for the recruitment of a later splicing factor or, as the H2B ubiquitination cycle regulates PolII passage through a gene [66], [81], it is possible that disruption of this cycle causes a subtle alteration of spliceosome dynamics that is not observable by ChIP. Furthermore, we cannot rule out the possibility that Bre1 has a ubiquitination target within the spliceosome or even ubiquitinates Npl3 itself.

Both splicing and mRNA processing are largely co-transcriptional processes in eukaryotes, from yeast [2] to human [82]–[84]. Our survey of *NPL3* genetic interactions has revealed a multitude of chromatin-connected factors with potential links to splicing and mRNA processing; overall, these results are thus consistent with an “integrator” role for Npl3 in gene expression (Figure 6). Our data provide a basis for the further study of the coupling of SR/hnRNP-dependent mRNA processing and transcription within a chromatin context, and have led to the discovery of Npl3-dependent and independent roles for Bre1 and histone H2B ubiquitination in splicing.

## Materials and Methods

A list of strains used and further strain construction details are available in Table S6.

### Synthetic genetic array

Unless otherwise indicated, yeast were grown as described in [85]. The *npl3Δ::NatNT2* “magic marker” query strain used in the SGA was YTK232D, and was previously used in [26]. YTK232D was generated using techniques outlined in [86]. Briefly, the *NPL3* open reading frame was replaced with *NatNT2* via integration of a PCR product generated with primers (5′- TACTTTTGAAGGAATCAAAATTAAGCAATTACGCTAAAACCATAAGGATAACATGGAGGCCCAGAATACCC-3′) and (5′-GTTTTAAAACAATTCATATCTTTTGTTAATTTCTCCTTTTTTTTTCTCAACCAGTATAGCGACCAGCATTC-3′) into the SGA diploid strain [87]. The diploid was sporulated and the MATα *npl3Δ::NatNT2* query strain was isolated by tetrad dissection, followed by re-selection of magic markers on SD medium lacking leucine and arginine but containing canavanine, s-AEC, and clonNAT [SD - LEU/ARG+100 µg/mL canavanine+100 µg/mL S-(2-Aminoethyl)-L-cysteine hydrochloride+100 µg/mL clonNAT]. The *NPL3* deletion was confirmed by PCR, and by Western blot for the absence of Npl3 using an α-Npl3 antibody [63].

The Synthetic Genetic Array was performed as described in [24] with the following exceptions: Here the *npl3*Δ query strain (YTK232D) was mated to the MAT**a** KanMX-marked deletion collection (OpenBiosystems: www.openbiosystems.com; formerly Research Genetics, Huntsville, AL). The collection was arrayed in duplicate in 384-well colony format using automated pinning (Colony Arrayer) and grown at 30°C for 2 days. Mating was carried out at 30°C for 2 days. Sporulation was carried out at 30°C for 7 days. MAT**a** double mutants were selected on SD medium lacking histidine and arginine but containing canavanine, S-AEC, G418, and clonNAT [SD - HIS/ARG+100 µg/mL canavanine+100 µg/mL S-(2-Aminoethyl)-L-cysteine hydrochloride+150 µg/mL G418 and 100 µg/mL clonNAT]. Double mutant arrays were re-pinned in replicate and photographed after the following incubations: 30°C for 5 days, 37°C for 5 days, or at 16°C for 4 and again after 8 days. Photographs were visually inspected for growth at 16°C (to identify suppressive interactions) or lack of colony growth at 30°C or 37°C (to identify synthetic lethal interactions).

### Directed genetics

The *npl3*Δ::*NatNT2* strain used for directed genetics (YTK234D) was previously used in [7]. Unless otherwise indicated, YTK234D was crossed to a series of MAT**a** KanMX4-marked deletion strains; diploids were selected by plating on YPD plates+100 µg/mL clonNAT+150 µg/mL G418. Double mutants were isolated by tetrad dissection or random sporulation, as indicated in Table S6. All single mutants were validated by PCR for the knockout chromosome prior to crossing to YTK234D. The *HTB1-WT* (WHY334) and *htb1-K123R* (WHY326) strains contain *htb2*Δ::*HygX4l* and the indicated *htb1* allele as the sole copy of H2B (gifts from W. Hwang and H. Madhani). They were mated as above, except the diploid strains were selected on YPD+100 µg/mL hygromycin+100 µg/mL clonNAT. Because the *htb1* allele is unmarked, the final *npl3*Δ*HTB1* and *npl3*Δ*htb1K123R* strains were confirmed by sequencing the *HTB1* gene and Western blot for the Npl3 protein. Genetic interactions with the *bre1H665A* allele were analyzed using a set of plasmids provided by the Shilatifard lab [19], designed to complement a *bre1*Δ allele. Complementation was achieved by plasmid transformation into YM1740 (*bre1*Δ) or YTK391B (*npl3*Δ*bre1*Δ), which were maintained on SD -LEU plates.

The *bre1*Δ::*NatNT2* (EMy32) and *ubp*8Δ::*NatNT2* (EMy442) strains were created by replacement of the endogenous ORF with NatNT2, as described in [86]. These strains were subsequently mated to *nam8*Δ, *mud1*Δ, *mud2*Δ, *syf2*Δ, *and snu66*Δ (for *bre1*Δ), and *lea1*Δ (for *bre1*Δ and *ubp8*Δ) from the deletion collection and double mutants were isolated via tetrad dissection. For the rest of the *ubp8*Δ genetics, the *ubp8*Δ::*KanMX4* strain from the deletion collection was mated to MATα NAT-marked “magic marked” splicing factor deletion strains. These splicing factor deletion strains were made by replacing the KanMX-marked ORFs with NatNT2, followed by crossing to a “magic marked” wild-type (YTK609) to isolate “magic marked” NAT-marked MATα spores. The *ubp8*Δ::*KanMX4* strain was mated to each NAT-marked splicing factor deletion strain and MAT**a** double mutants were isolated by tetrad dissection followed by selection on SD medium lacking histidine and arginine but containing canavanine, S-AEC, G418, and clonNAT [SD - HIS/ARG+100 µg/mL canavanine+100 µg/mL S-(2-Aminoethyl)-L-cysteine hydrochloride+150 µg/mL G418 and 100 µg/mL clonNAT].

For individual growth assays, log-phase yeast were diluted to OD_600_ = 0.1, spotted onto YPD plates (unless specifically mentioned) in a 5-fold dilution series and grown at the indicated temperatures. For each cross, growth of the double mutant was confirmed for ≥2 double mutant isolates, and a representative isolate is shown. The single mutants and wild-type strains shown are either parental strains, or were re-isolated from tetra-type tetrads. The *bre1H665A* and *BRE1* strains were serially diluted onto SD –LEU plates.

### Process and complex analyses

We sought to integrate the diverse sources of genetic interaction information available to us in order to create a comprehensive dataset for statistical analyses. Because the stronger synthetic interactions were identified in the 30°C SGA, we began with this list of genes whose deletion caused lethality in combination with *npl3*Δ (see Table S1 – 30°C) and added genes identified as causing markedly decreased growth, as gauged by serial dilution, or lethality, as gauged by loss of double mutant spore after tetrad dissection (Figure 1C, 1D, 1E and Table S1). We further added to this list genes identified as synthetic sick or lethal in the E-MAP [26] *i.e.*, having a genetic interaction score of ≤−2.5.

Biological process definitions were obtained from the Gene Ontology annotations maintained at SGD [88] on April 15th 2012. Forty-five high-level (GO Slim) terms were used and are included in Table S7. Protein complex definitions were obtained from a manually curated list, CYC2008 [89], and augmented with the RAD6 Complex (*RAD6*, *BRE1*, *LGE1*), which was not annotated when the list was created. A hypergeometric test was used to identify complexes and processes that were significantly enriched with genetic interactions. Complex enrichment p-values were corrected for multiple testing using the empirical re-sampling method of Berriz *et al.* [90] (as 409 complexes were assessed for enrichment), while process enrichment p-values were corrected for using the simpler Bonferoni correction. The results of the these analyses are included in Table S2 (by process) and Table S3 (by complex). The network diagram in Figure 1B was drawn using Cytoscape [91]. For Figure 1B, complexes were referred to by their more common names. The Figure 2A diagram was created to highlight a subset of suppressive interactions identified in the 16°C SGA and the full list of suppressors is available in Table S4.

### Co-immunoprecipitation

Co-immunoprecipitation assays were performed as in [7] with extracts from the indicated GFP-tagged or HA-tagged strains. The Nup188-HA strain contains a plasmid encoding Nup188-3XHA. The other strains were tagged endogenously. Briefly, samples were separated by 10% SDS-PAGE and probed by Western blot with either monoclonal α-GFP (Roche 1814460), α-HA (12CA5; Roche 11583816001), or polyclonal α-Npl3 antibodies [63]. Total samples equivalent to 1/60^th^ of the input were analyzed in parallel.

### Microarrays

Cultures were grown according to standard techniques [85] in rich medium supplemented with 2% glucose. Strains were cultured overnight to saturation and diluted to OD_600_ = 0.1 in the morning. The strains were allowed to grow at 30°C until reaching mid-log phase (OD_600_ = 0.5–0.7), at which point they were collected (for Figure S6), or rapidly shifted to either 37°C for 30 minutes or 16°C for 2.5 hours, as indicated. Cultures were collected by centrifugation and snap frozen in liquid nitrogen. Total cellular RNA was isolated using hot acid phenol followed by isopropanol precipitation, as outlined in [92] but with modifications detailed in [93]. cDNA from each strain was synthesized, and labeled with Cy3 or Cy5 according to the low-throughput sample preparation method described in [65].

The optimized oligos listed in [65] were robotically arrayed onto poly-L-lysine coated glass slides (slides from ThermoScientific C40-5257-M20) and slides were processed using the protocols detailed in [65], [94] Each biological replicate contains 6 technical replicates for each feature as well as dye-flipped replicates. Microarrays were scanned using Axon Instruments GenePix 4000B at 635 nm and 532 nm wavelengths and image analysis was done using Axon Instruments GenePix Pro version 5.1. Spots were manually removed from analysis if they contained obvious defects or uncharacteristically high background; the ratio of the median intensities for 535 nm and 625 nm was calculated for each remaining spot. Technical replicate spots and dye flipped replicates were combined and normalized as in [65]. The resulting log_2_-transformed values for each feature were averaged over 2–5 biological replicates. Averaged data were subjected to hierarchical clustering using average linkage, and uncentered Pearson correlation as the similarity metric using Cluster 3.0 [95]. Resulting heat maps in Figure 4, Figure S2, and Figure S6 were created using Java Treeview [96]. To normalize for changes in total expression evident in the microarrays, Intron Accumulation Indices (IAI) were calculated for each intron containing gene as in [69]; specifically, we calculated log_2_(Intron_mutant_/Intron_WT_)-log_2_(Exon_mutant_/Exon_WT_) for each gene. The IAI heat map is shown in Figure S2. These values were converted into a histogram for Figure 4 using the following cutoffs: −0.3≥IAI≥0.3.

### Dt50 Fluorescent *In Situ* Hybridization

The dT50 assay was performed based on the protocol outlined in [97] with the following modifications. Specifically, 2 mL cultures were fixed in 5% formaldehyde for 1.5 hours after having reached OD_600_ = 0.2–0.3. Cells were washed 4 times in wash buffer (100 mM Potassium Phosphate, 1.2 M Sorbitol) before a 40-minute treatment with 27 µg zymolyase at 37°C. An additional fixation was performed in 8% paraformaldehyde in PBS+10 mM MgCl2 and spheroplasted cells were applied to poly-lysine-treated chamber slides (LabTek 178599). Attached cells were treated with ice-cold methanol (−20°C) and allowed to dry. Hybridization to digoxin-conjugated dT50 oligo in blocking buffer was performed at 37°C overnight. Chambers were washed with 2× (20 minutes), 1× (20 minutes) and 0.5× SSC (10 minutes at 37°C) before a 30-minute incubation with FITC- conjugated anti-Digoxin Fab fragments (Roche 1207741) in blocking buffer (1∶25 dilution, 37°C). Antibody was aspirated and three 5-minute washes of PBS +10 mM MgCl2 were performed. Chambers were treated with 0.5 mg/mL DAPI for 2 minutes and slides were mounted using ProLong Gold Antifade Reagent (Invitrogen P36934) according to manufacturer instructions. Slides were visualized using an Olympus BX60 microscope equipped with FITC HiQ and DAPI HiQ Filters (Chroma Technology Corporation). The assay was performed on two biological replicates and representative images are shown. Specificity of the probe and FITC labeling was determined by incubation with hybridization mix lacking probe (data not shown).

### *Sus1* Splicing Assay

*SUS1* splicing efficiency was measured essentially according to the non-radioactive protocol described in [74]. Specifically, 10 µg RNA from cultures grown at 30°C was treated with DNaseI (Promega) and RNA was converted to cDNA using 1 µg *SUS1* Reverse primer [70]. cDNAs were diluted 1∶200 and 10 µL was used in a 25 µL PCR (BioRad iProof) with *SUS1*-specific primers [70]. 25 cycles of PCR were performed and the resulting products were separated on an 8% polyacrylamide gel. Gels were stained using SybrGold and bands were quantified using an AlphaImager HP camera and software. 2–3 technical replicates of 2 biological samples were performed. Shown are a representative gel and the average and standard deviations of all technical replicates. A no-Reverse Transcriptase control was performed for each sample and none showed amplification (data not shown).

### H2b Ubiquitination Western Blot

A TCA precipitation was performed on strains grown at 30°C [98] and samples were run on a 15% SDS polyacrylamide gel and transferred to PVDF membrane. Membrane was blocked using Li-Cor blocking buffer, followed by incubation of a 1∶1000 dilution of α-H2B antibody (Active Motif 39237) overnight at 4°C. Visualization of bands was achieved with a secondary antibody conjugated to infrared dye (LI-COR 926-32211). The membrane was scanned using the LI-COR Odyssey scanner and software. Shown is a representative Western blot. The assay was performed with 3 biological replicates and shown are the average and standard deviation of the three replicates.

### Qpcr Assay

RNA was extracted as described above from strains grown under the same conditions as for the microarray experiment. Five µg RNA were treated with DNaseI (Promega) before being primed with random 9-mers and reverse transcribed. Samples were diluted as necessary and 10 µL were used in each qPCR. qPCRs were run on a C1000 ThermoCycler (BioRad) with an annealing temperature of 55°C. Each qPCR run was finished with a melt curve to determine the homogeneity of the amplified product. Starting quantity was calculated using a standard curve for each primer set. 2–4 technical replicates were performed for 1–5 biological replicates. Error bars represent standard deviation for biological replicates. For samples with 1 biological replicate, standard deviation of technical replicates is shown with uncapped error bars (Figure S3). A no-Reverse Transcriptase control was also generated for each RNA sample and these samples yielded negligible amplification (data not shown). Primers used in the qPCR are listed in Table S8. Each gene was measured using intron- and exon- specific primer sets. The Intron/Exon ratio for each mutant was normalized to its corresponding wild-type before averaging.

## Supporting Information

Figure S1 *NPL3* does not affect global H2B ubiquitination levels. (A) Western blot analysis of histone H2B ubiquitination levels. Whole cell extracts from the indicated strains were subjected to electrophoresis to separate the ubiquitinated H2B from unmodified H2B, followed by Western blotting using α-H2B antibody. Shown is a representative blot. (B) Quantitation of H2B ubiquitination levels. Shown are the average percentages of ubiquitinated H2B from the indicated strains. Error bars represent standard deviation of three biological replicates.
(TIF)

Figure S2 Heat map representation of the Intron Accumulation Indices used to generate histogram in Figure 4. Shown are log_2_-based Intron Accumulation Index scores for each intron-containing gene, generated by normalizing fold intron changes to fold exon changes (see Materials and Methods for details). Genotype of each strain measured is listed above the heat maps. Transcripts that encode the ribosomal protein genes (RPGs) are highlighted in purple to the right of the heat maps. Gene order along the y-axis is the same for all genotypes.
(TIF)

Figure S3 qPCR validation of the microarrays. (A) Shown are log_2_-based Intron Accumulation Index scores for the indicated genes, as reproduced from Figure S2. The genotype of each strain is listed above the heat map. *K123R* refers to the *htb1K123R* allele.(B) RT-qPCR measurements of un-spliced mRNAs using single-locus RT-qPCR. Percent un-spliced RNA was calculated for each mutant and is represented as fold change compared to wild-type. Capped error bars represent standard deviation of biological replicates; uncapped error bars represent standard deviation of qPCR replicates.
(TIF)

Figure S4 *SUS1* splicing is not sensitive to deletion of *NPL3*. (A) Analysis of *SUS1* splicing using quantitative PCR on cDNA generated from the indicated strains. To the right of the gel, is a schematic of the *SUS1* gene and the predicted mobility of its un-spliced, partially spliced, and fully spliced isoforms. Arrows indicate position of primers used in the PCR. Shown is a representative gel. (B) Quantitation of *SUS1* PCR. To obtain the data shown in panel B, we took the average of two technical replicates generated from two separate biological samples. Error bars represent standard deviation of all replicates.
(TIF)

Figure S5 *NPL3* and *BRE1* do not affect bulk mRNA export. *In situ* hybridization of poly-dT oligo shows whole cell localization of mRNAs in wild-type, *npl3*Δ, *bre1*Δ, *and npl3*Δ*bre1*Δ strains. The positive control *thp1*Δ strain is included to show nuclear localization coincident with DAPI (nuclear) staining.
(TIF)

Figure S6 Spicing is not sensitive to mutation of the Npl3 phosphorylation site. (A) Splicing profiles of the *npl3S411A* and *npl3*Δ strains grown at 30°C. The heat map shows the log_2_-ratio for each gene feature of the indicated strain compared to wild-type. Gene order along the y-axis is the same for all arrays. Transcripts that encode the ribosomal protein genes (RPGs) are highlighted in purple to the right of the heat maps. (B) Histogram shows the number of genes with a log_2_-based Intron Accumulation Index score greater than 0.3 for the *npl3S411A* and *npl3*Δ strains. Heat map within histogram bars shows distribution of the severity of splicing defect.
(TIF)

Table S1 Genes whose deletion caused severe synthetic sickness or lethality with *npl3*Δ. List of genes whose deletion caused severe synthetic sickness or lethality with *npl3*Δ at 30°C or 37°C. Included are the systematic and common names, the synthetic genetic array in which each strain was identified, and the putative function as annotated by SGD (yeastgenome.org). Phenotype of the genetic interactions validated using directed genetics is listed under “notes”. Any strains added using directed genetics are listed at the bottom of the table. Genes are sorted first by screen in which they were identified, and then alphabetically by common name.
(XLS)

Table S2 GO-term analysis by Biological Process. Results of GO-term analysis by “Biological Process” with the associated p-values (pre- and post-correction). Biological Process definitions are available in Table S7.
(XLSX)

Table S3 Protein Complex enrichment analysis. Results of protein complex enrichment analysis with the associated p-values (pre- and post-correction for multiple testing). Significantly enriched complexes in bold are shown in Figure 1B using their more common names. Protein complex definitions were obtained from [89] with the addition of the RAD6 Complex containing *RAD6*, *BRE1* and *LGE1*, which was not annotated at the time.
(XLSX)

Table S4 Genes whose deletion allowed growth in an *npl3*Δ strain. List of genes whose deletion allowed growth in an *npl3*Δ strain in the 16°C synthetic genetic array. Included are the systematic and common names, as well as the time-point at which growth was visualized, and the putative function of each gene. Any strains validated using directed genetics (by tetrad analyses or random sporulation) are indicated. Genes are sorted first by interval of time necessary to observe growth, and then alphabetically by common name.
(XLS)

Table S5 Averaged microarray results used to generate heat map in Figure 4. Listed are log_2_ ratios of the intensities for each gene feature for the indicated strains compared to wild-type. “_2” refers to the second intron. Also included are the Intron Accumulation Index scores for each gene used to generate the heat map in Figure S2.
(XLS)

Table S6 Strains used in this manuscript, and more detailed methods for their generation.
(PDF)

Table S7 Biological Process definitions used to categorize *npl3*Δ genetic interactions.
(XLSX)

Table S8 Primers used in the qPCR assay.
(PDF)
