# Supplementary figures and images for: The Yeast SR-Like Protein Npl3 Links Chromatin Modification to mRNA Processing
Source: PLoS Genet. 2012 Nov 29;8(11):e1003101. doi: 10.1371/journal.pgen.1003101 (PMC3510044; doi:10.1371/journal.pgen.1003101)

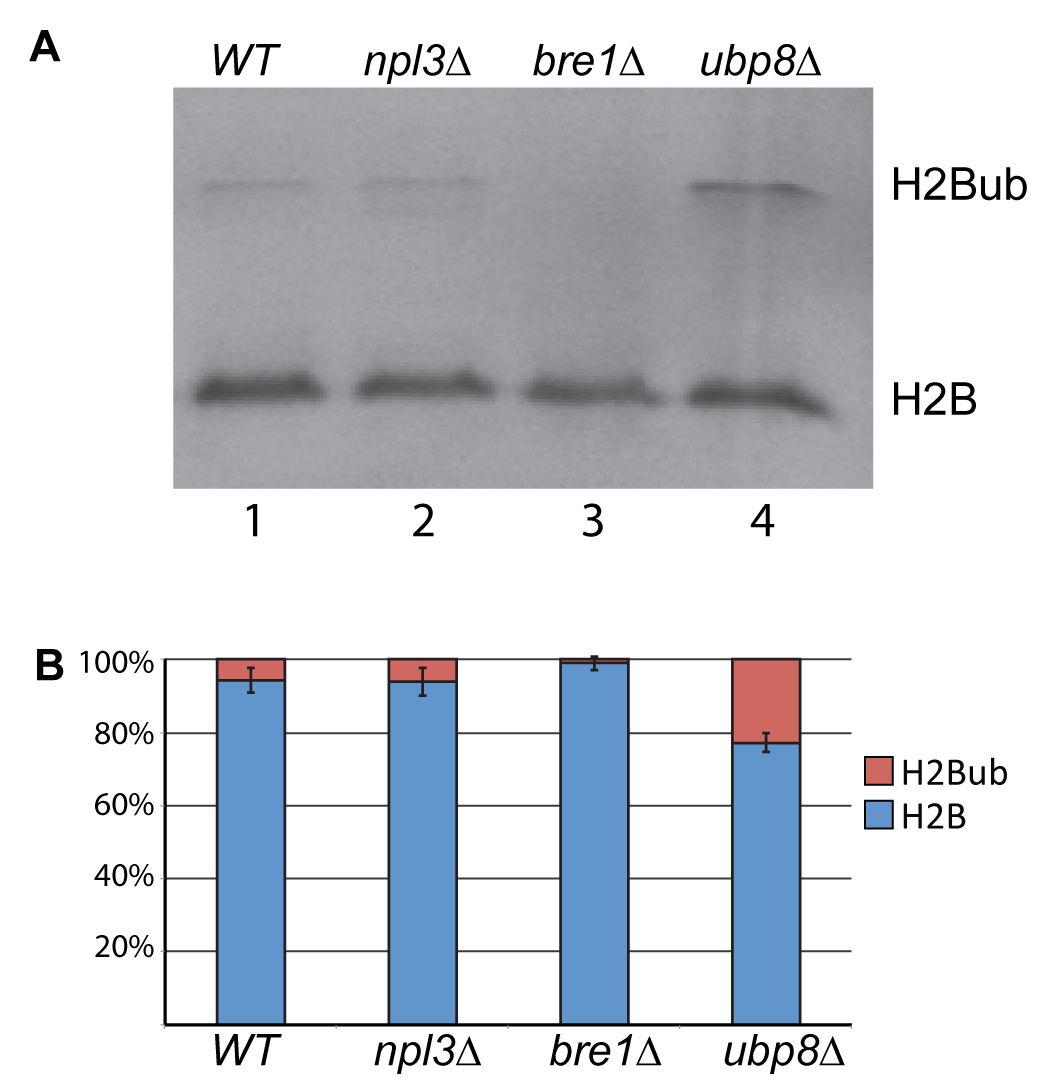

Supplement: Figure S1 — NPL3 does not affect global H2B ubiquitination levels. (A) Western blot analysis of histone H2B ubiquitination levels. Whole cell extracts from the indicated strains were subjected to electrophoresis to separate the ubiquitinated H2B from unmodified H2B, followed by Western blotting using α-H2B antibody. Shown is a representative blot. (B) Quantitation of H2B ubiquitination levels. Shown are the average percentages of ubiquitinated H2B from the indicated strains. Error bars represent standard deviation of three biological replicates. (TIF) [file pgen.1003101.s001.tif]

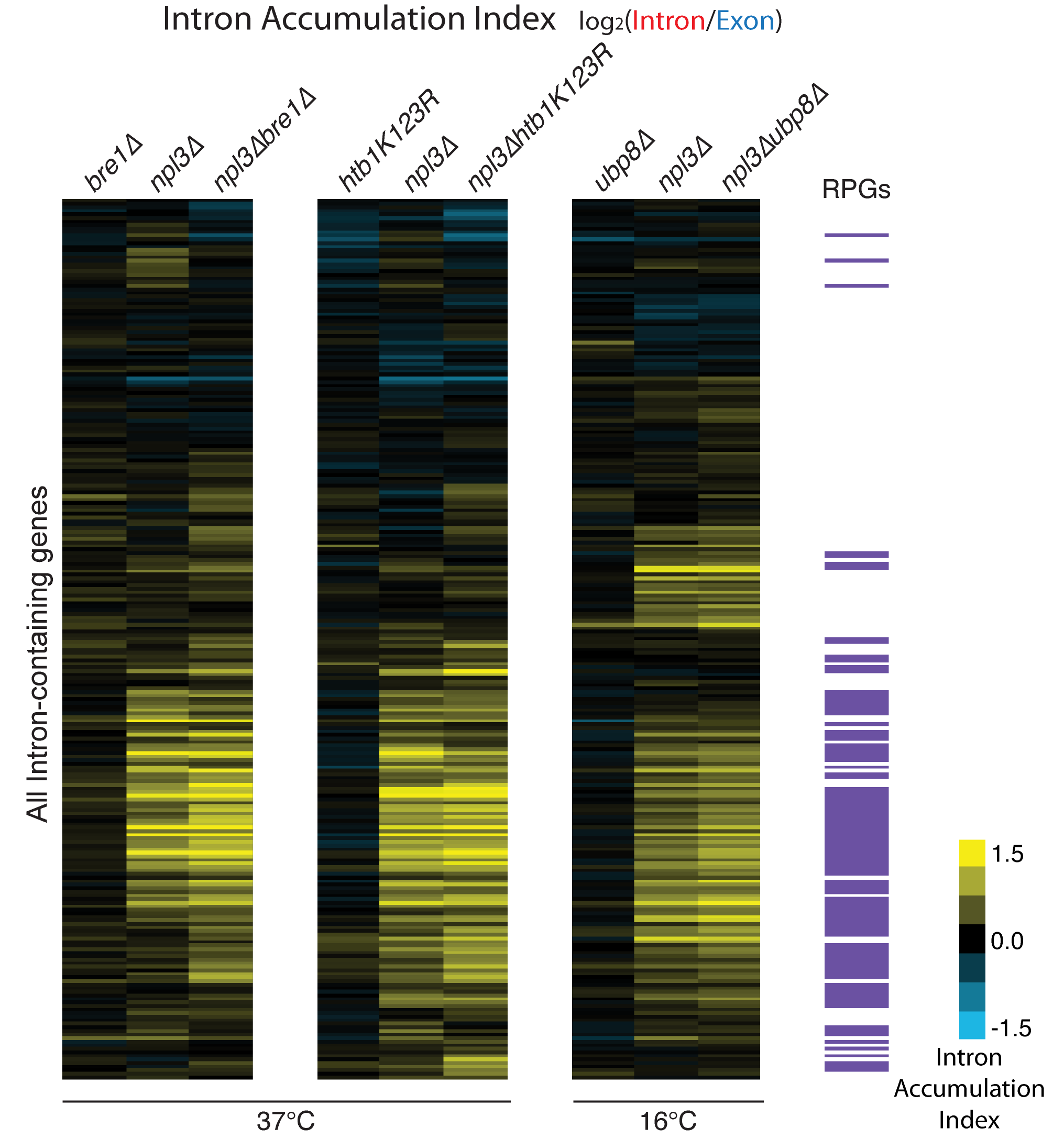

Supplement: Figure S2 — Heat map representation of the Intron Accumulation Indices used to generate histogram in Figure 4. Shown are log2-based Intron Accumulation Index scores for each intron-containing gene, generated by normalizing fold intron changes to fold exon changes (see Materials and Methods for details). Genotype of each strain measured is listed above the heat maps. Transcripts that encode the ribosomal protein genes (RPGs) are highlighted in purple to the right of the heat maps. Gene order along the y-axis is the same for all genotypes. (TIF) [file pgen.1003101.s002.tif]

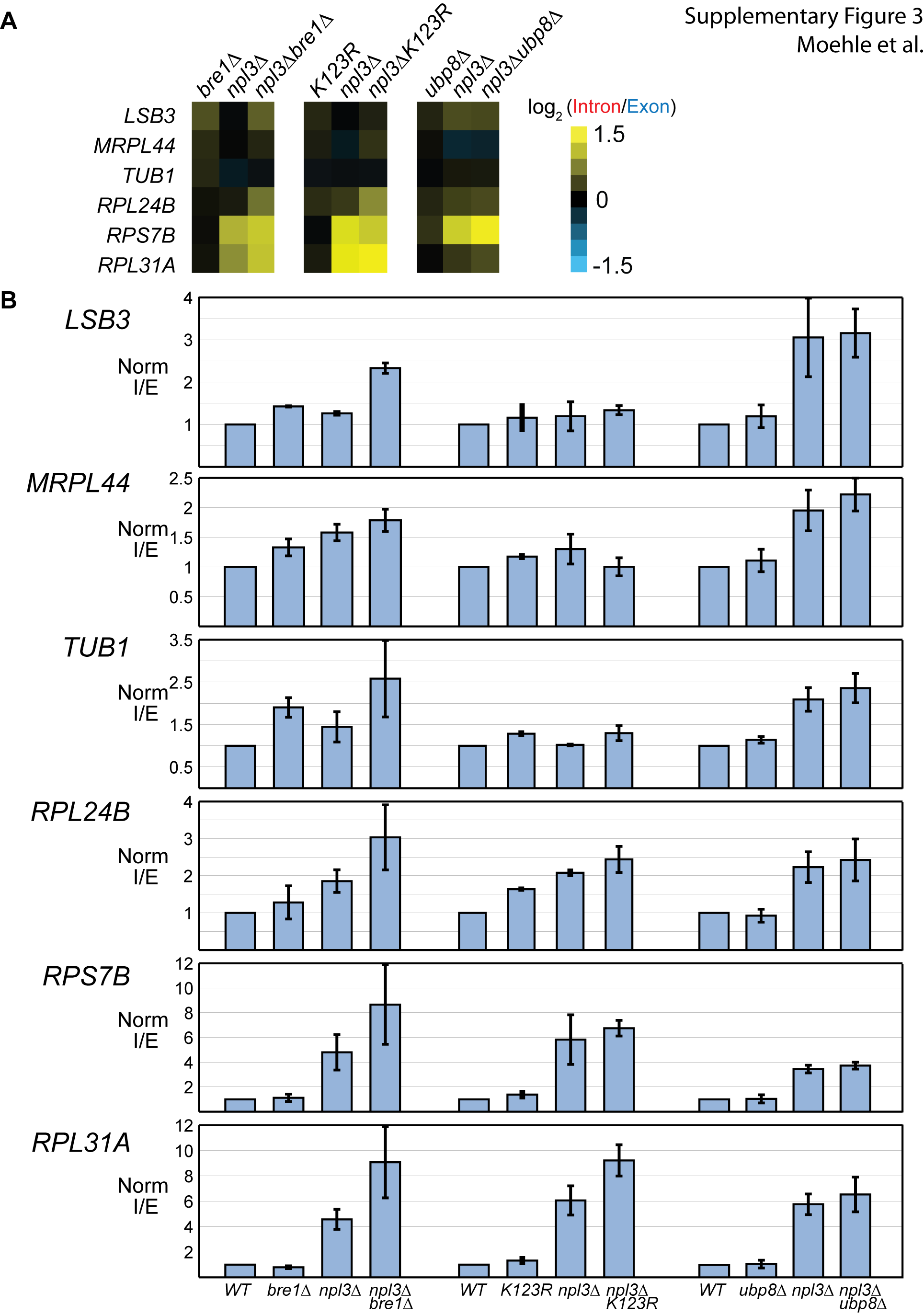

Supplement: Figure S3 — qPCR validation of the microarrays. (A) Shown are log2-based Intron Accumulation Index scores for the indicated genes, as reproduced from Figure S2. The genotype of each strain is listed above the heat map. K123R refers to the htb1K123R allele.(B) RT-qPCR measurements of un-spliced mRNAs using single-locus RT-qPCR. Percent un-spliced RNA was calculated for each mutant and is represented as fold change compared to wild-type. Capped error bars represent standard deviation of biological replicates; uncapped error bars represent standard deviation of qPCR replicates. (TIF) [file pgen.1003101.s003.tif]

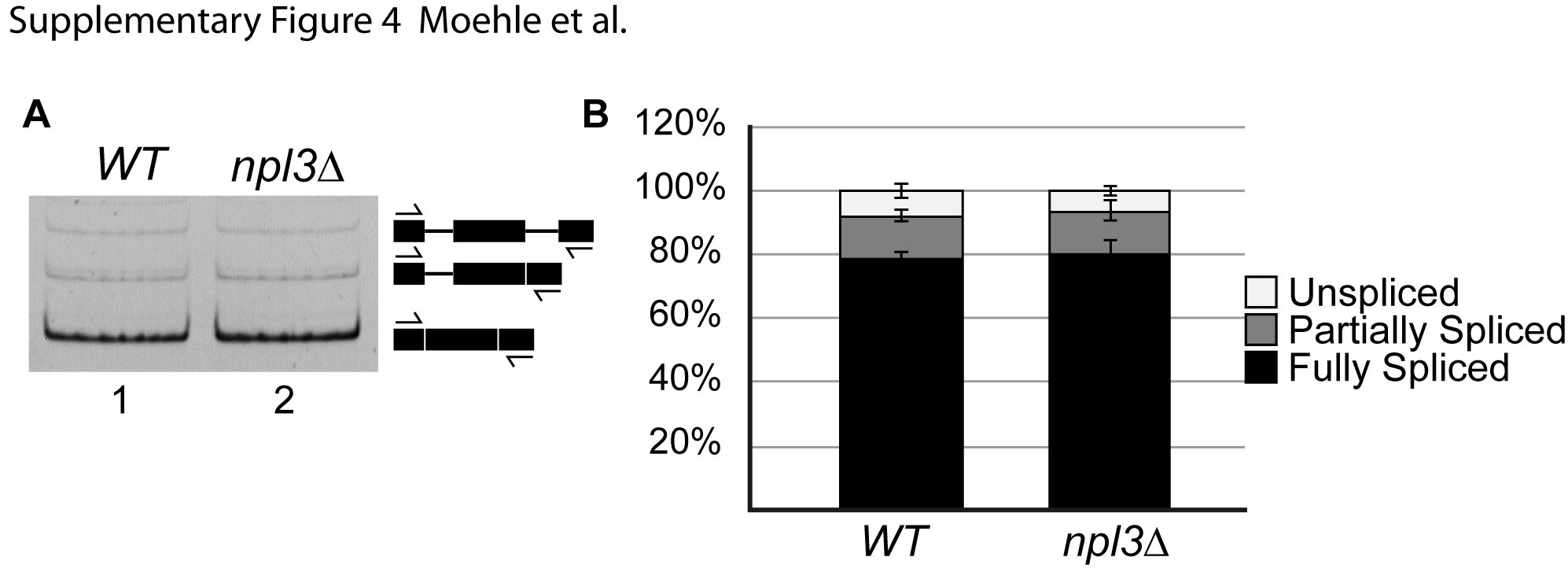

Supplement: Figure S4 — SUS1 splicing is not sensitive to deletion of NPL3. (A) Analysis of SUS1 splicing using quantitative PCR on cDNA generated from the indicated strains. To the right of the gel, is a schematic of the SUS1 gene and the predicted mobility of its un-spliced, partially spliced, and fully spliced isoforms. Arrows indicate position of primers used in the PCR. Shown is a representative gel. (B) Quantitation of SUS1 PCR. To obtain the data shown in panel B, we took the average of two technical replicates generated from two separate biological samples. Error bars represent standard deviation of all replicates. (TIF) [file pgen.1003101.s004.tif]

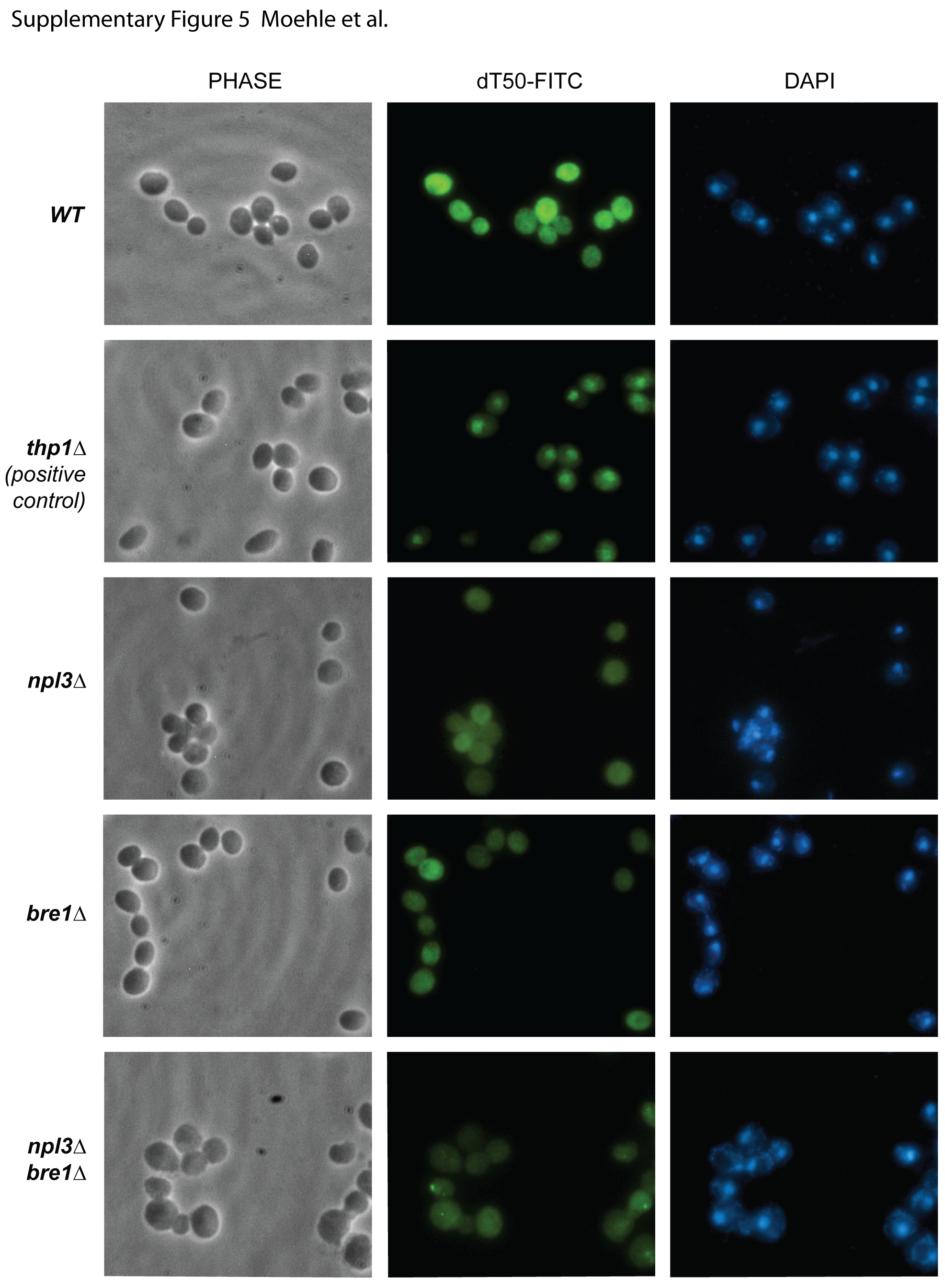

Supplement: Figure S5 — NPL3 and BRE1 do not affect bulk mRNA export. In situ hybridization of poly-dT oligo shows whole cell localization of mRNAs in wild-type, npl3Δ, bre1Δ, and npl3Δbre1Δ strains. The positive control thp1Δ strain is included to show nuclear localization coincident with DAPI (nuclear) staining. (TIF) [file pgen.1003101.s005.tif]

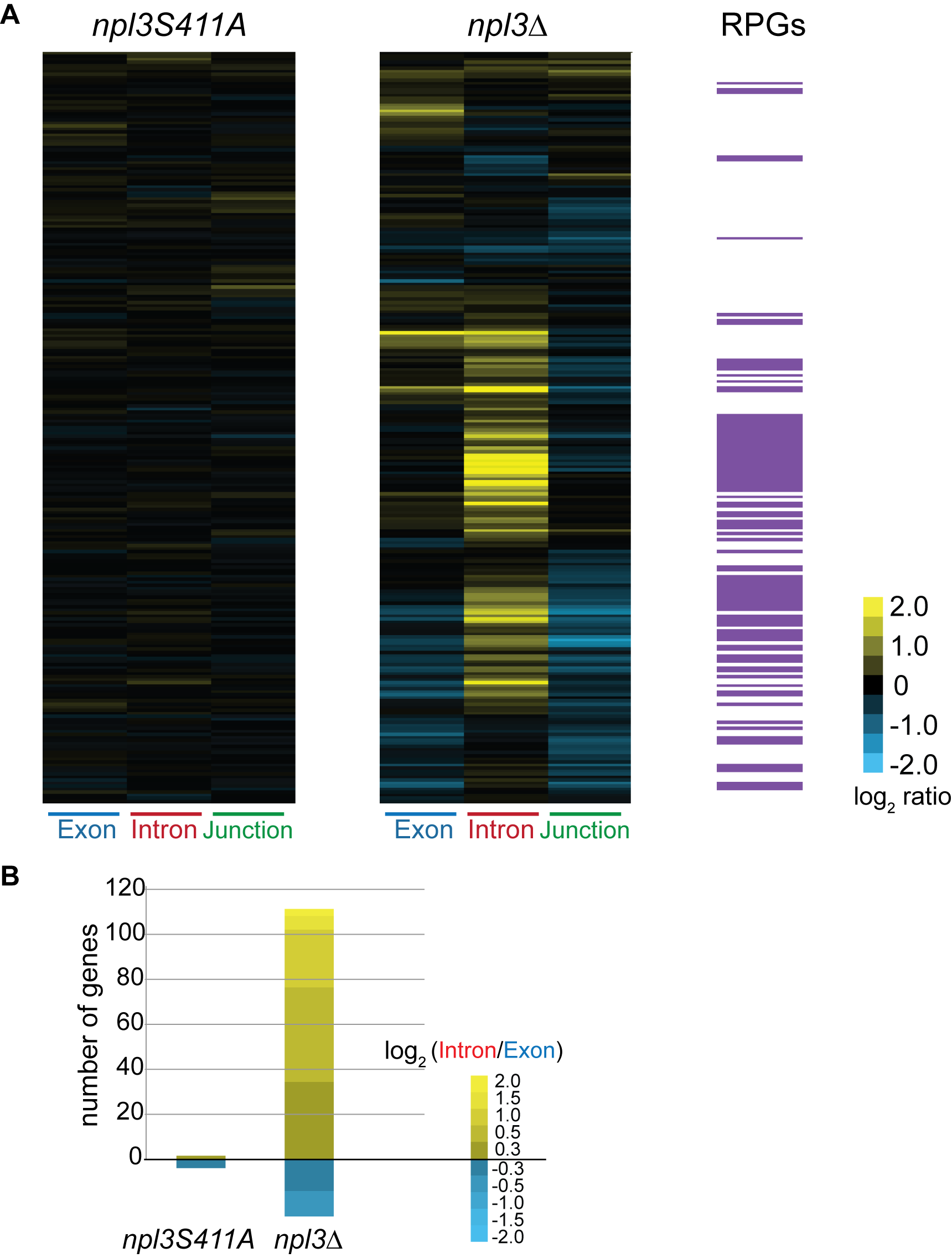

Supplement: Figure S6 — Spicing is not sensitive to mutation of the Npl3 phosphorylation site. (A) Splicing profiles of the npl3S411A and npl3Δ strains grown at 30°C. The heat map shows the log2-ratio for each gene feature of the indicated strain compared to wild-type. Gene order along the y-axis is the same for all arrays. Transcripts that encode the ribosomal protein genes (RPGs) are highlighted in purple to the right of the heat maps. (B) Histogram shows the number of genes with a log2-based Intron Accumulation Index score greater than 0.3 for the npl3S411A and npl3Δ strains. Heat map within histogram bars shows distribution of the severity of splicing defect. (TIF) [file pgen.1003101.s006.tif]
