# Supplementary material for: The Yeast SR-Like Protein Npl3 Links Chromatin Modification to mRNA Processing
Source: PLoS Genet. 2012 Nov 29;8(11):e1003101. doi: 10.1371/journal.pgen.1003101 (PMC3510044; doi:10.1371/journal.pgen.1003101)
Supplement: Table S6 — Strains used in this manuscript, and more detailed methods for their generation. (PDF) [file pgen.1003101.s012.pdf]

Supplementary Table 6.
Yeast Strains used in Moehle *et al.* "The Yeast SR-like Protein Npl3 Links Chromatin Modification to mRNA processing"

**Strain**	**Genotype**	**Notes**
YTK59	*MAT***a** *his3Δ1 leu2Δ0 met15Δ0 ura3Δ0*	A
YTK219	*MAT***a***/α; his3Δ1/his3Δ1 leu2Δ0/leu2Δ0 LYS2/lys2 met15Δ0/met15Δ0 uraΔ0/uraΔ0 can1Δ::STE2pr-SpHIS5/CAN1 lyp1Δ::STE3pr-LEU2/LYP1 CHY2/chy2*	B
YTK232D	*npl3Δ::NatNT2 MATα; leu2Δ0 met15Δ0 ura3Δ0 can1Δ::STE2pr-SpHIS5 lyp1Δ::STE3pr-LEU2 chy2*	C
YTK234D	*npl3Δ::NatNT2 MATα his3Δ1 leu2Δ0 lys2Δ0 ura3Δ0*	C
N/A	*X::KanMX4 MAT***a** *his3Δ1 leu2Δ0 ura3Δ0 met15Δ0*	D
YM1740	*bre1Δ::KanMX4 MAT***a** *his3Δ1 leu2Δ0 ura3Δ0 met15Δ0*	E
YM1739	*rad6Δ::KanMX4 MAT***a** *his3Δ1 leu2Δ0 ura3Δ0 met15Δ0*	E
YM1741	*lge1Δ::KanMX4 MAT***a** *his3Δ1 leu2Δ0 ura3Δ0 met15Δ0*	E
EMy5	*paf1Δ::KanMX4 MAT***a** *his3Δ1 leu2Δ0 lys2Δ0 ura3Δ0*	F
EMy8	*htz1Δ::KanMX4 MAT***a** *his3Δ1 leu2Δ0 lys2Δ0 ura3Δ0*	F
EMy11	*swr1Δ::KanMX4 MAT***a** *his3Δ1 leu2Δ0 lys2Δ0 ura3Δ0*	F
EMy14	*vps72Δ::KanMX4 MAT***a** *his3Δ1 leu2Δ0 lys2Δ0 ura3Δ0*	F
EMy456	*set1Δ::KanMX4 MAT***a** *his3Δ1 leu2Δ0 lys2Δ0 ura3Δ0*	F
YTK391B	*npl3Δ::NatNT2 bre1Δ::KanMX4 his3Δ1 leu2Δ0 ura3Δ0*	G
EMy415	*npl3Δ::NatNT2 rad6Δ::KanMX4 his3Δ1 leu2Δ0 ura3Δ0*	G
YTK537A	*npl3Δ::NatNT2 cdc73Δ::KanMX4 his3Δ1 leu2Δ0 ura3Δ0*	G
YTK538A	*npl3Δ::NatNT2 leo1Δ::KanMX4 his3Δ1 leu2Δ0 ura3Δ0*	G
YTK551A	*npl3Δ::NatNT2 spt3Δ::KanMX4 his3Δ1 leu2Δ0 ura3Δ0*	G
YTK552A	*npl3Δ::NatNT2 spt8Δ::KanMX4 his3Δ1 leu2Δ0 ura3Δ0*	G
YTK554A	*npl3Δ::NatNT2 ada2Δ::KanMX4 his3Δ1 leu2Δ0 ura3Δ0*	G
EMy460	*npl3Δ::NatNT2 set1Δ::KanMX4 his3Δ1 leu2Δ0 lys2Δ0 ura3Δ0*	G
YTK736A	*npl3Δ::NatNT2 spp1Δ::KanMX4 his3Δ1 leu2Δ0 ura3Δ0*	G
YTK737A	*npl3Δ::NatNT2 sdc1Δ::KanMX4 his3Δ1 leu2Δ0 ura3Δ0*	G
YTK550A	*npl3Δ::NatNT2 rad18Δ::KanMX4 his3Δ1 leu2Δ0 ura3Δ0*	G
YTK245A	*npl3Δ::NatNT2 rrp6Δ::KanMX4 his3Δ1 leu2Δ0 ura3Δ0*	G
YTK429A	*npl3Δ::NatNT2 set3Δ::KanMX4 his3Δ1 leu2Δ0 ura3Δ0*	G
YTK533A	*npl3Δ::NatNT2 ubp8Δ::KanMX4 his3Δ1 leu2Δ0 ura3Δ0*	G
YTK534A	*npl3Δ::NatNT2 sgf11Δ::KanMX4 his3Δ1 leu2Δ0 ura3Δ0*	G
YTK303	*npl3Δ::NatNT2 sgf29Δ::KanMX4 his3Δ1 leu2Δ0 ura3Δ0*	G
YTK749A	*npl3Δ::NatNT2 ctk1Δ::KanMX4 his3Δ1 leu2Δ0 ura3Δ0*	G
YTK535B	*npl3Δ::NatNT2 set2Δ::KanMX4 his3Δ1 leu2Δ0 ura3Δ0*	G
YTK557A	*npl3Δ::NatNT2 eaf3Δ::KanMX4 his3Δ1 leu2Δ0 ura3Δ0*	G
YTK333	*npl3Δ::NatNT2 rco1Δ::KanMX4 his3Δ1 leu2Δ0 ura3Δ0*	H
YTK553A	*npl3Δ::NatNT2 chd1Δ::KanMX4 his3Δ1 leu2Δ0 ura3Δ0*	G
YTK735A	*npl3Δ::NatNT2 ckb2Δ::KanMX4 his3Δ1 leu2Δ0 ura3Δ0*	G
YTK558A	*npl3Δ::NatNT2 eaf7Δ::KanMX4 his3Δ1 leu2Δ0 ura3Δ0*	G
YTK348	*npl3Δ::NatNT2 dst1Δ::KanMX4 his3Δ1 leu2Δ0 ura3Δ0*	G
YTK315	*npl3Δ::NatNT2 hst1Δ::KanMX4 his3Δ1 leu2Δ0 ura3Δ0*	H
YTK331	*npl3Δ::NatNT2 tho2Δ::KanMX4 his3Δ1 leu2Δ0 ura3Δ0*	H
WHY334	*"HTB1" HTB1 htb2Δ::hygX4l MAT***a** *his3Δ leu2Δ ura3Δ (note: wild-type strain for WHY326)*	I

WHY326	*"htb1K123R" htb1K123R htb2Δ::hygX4l MAT***a** *his3Δ leu2Δ ura3Δ*	I
YTK396A	*"npl3ΔHTB1" HTB1 htb2Δ::hygX4l npl3Δ::NatNT2 his3Δ0 leu2Δ0 ura3Δ0*	J
YTK397A	*"npl3Δhtb1K123R" htb1K123R htb2Δ::hygX4l npl3Δ::NatNT2 his3Δ leu2Δ ura3Δ*	J
YTK572	*H3K36A" hht2K4A-HHF2-URA3 hht1-hhf1::NatMX MAT***a** *his3Δ200 leu2Δ1 lys2Δ0 met15Δ0 trp1Δ63 ura3-167 ade2::hisG RDN1::Ty1-MET15 TelV::ADE2*	K
YTK575	*H3K36A" hht2K36A-HHF2-URA3 hht1-hhf1::NatMX MAT***a** *his3Δ200 leu2Δ1 lys2Δ0 met15Δ0 trp1Δ63 ura3-167 ade2::hisG RDN1::Ty1-MET15 TelV::ADE2*	K
YTK576	"H3K79A" *hht2K79A-HHF2-URA3 hht1-hhf1::NatMX MAT***a** *his3Δ200 leu2Δ1 lys2Δ0 met15Δ0 trp1Δ63 ura3-167 ade2::hisG RDN1::Ty1-MET15 TelV::ADE2*	K
EMy99	*npl3Δ::KanMX4 hht2K4A-HHF2-URA3 hht1-hhf1::NatMX MAT***a** *his3Δ200 leu2Δ1 lys2Δ0 met15Δ0 trp1Δ63 ura3-167 ade2::hisG RDN1::Ty1-MET15 TelV::ADE2*	L
EMy94	*npl3Δ::KanMX4 hht2K36A-HHF2-URA3 hht1-hhf1::NatMX MAT***a** *his3Δ200 leu2Δ1 lys2Δ0 met15Δ0 trp1Δ63 ura3-167 ade2::hisG RDN1::Ty1-MET15 TelV::ADE2*	L
EMy102	*npl3Δ: KanMX4 hht2K79A-HHF2-URA3 hht1-hhf1::NatMX MAT***a** *his3Δ200 leu2Δ1 lys2Δ0 met15Δ0 trp1Δ63 ura3-167 ade2::hisG RDN1::Ty1-MET15 TelV::ADE2*	L
YTK609	*MATα Magic Marker strain his3Δ1 leu2Δ0 LYS2+met15Δ0 ura3Δ0 can1Δ::STE2pr-SpHIS5 lyp1Δ::STE3pr-LEU2*	M
YTK660A	*mud1Δ::NatNT2 MATα can1Δ::STE2pr-SpHIS5 lyp1Δ::STE3pr-LEU2 his3Δ1 leu2Δ0 uraΔ0*	N
YTK661	*nam8Δ::NatNT2 MATα can1Δ::STE2pr-SpHIS5 lyp1Δ::STE3pr-LEU2 his3Δ1 leu2Δ0 uraΔ0*	N
YTK662A	*cus2Δ::NatNT2 MATα can1Δ::STE2pr-SpHIS5 lyp1Δ::STE3pr-LEU2 his3Δ1 leu2Δ0 uraΔ0*	N
YTK663A	*mud2Δ::NatNT2 MATα can1Δ::STE2pr-SpHIS5 lyp1Δ::STE3pr-LEU2 his3Δ1 leu2Δ0 uraΔ0*	N
YTK664A	*isy1Δ::NatNT2 MATα can1Δ::STE2pr-SpHIS5 lyp1Δ::STE3pr-LEU2 his3Δ1 leu2Δ0 uraΔ0*	N
YTK649	*snu66Δ::NatNT2 MATα can1Δ::STE2pr-SpHIS5 lyp1Δ::STE3pr-LEU2 his3Δ1 leu2Δ0 uraΔ0*	N
YTK722A	*mud1Δ::NatNT2 ubp8Δ::KanMX4 MAT***a** *can1Δ::STE2pr-SpHIS5 lyp1Δ::STE3pr-LEU2 his3Δ1 leu2Δ0 uraΔ0*	O
YTK723A	*nam8Δ::NatNT2 ubp8Δ::KanMX4 MAT***a** *can1Δ::STE2pr-SpHIS5 lyp1Δ::STE3pr-LEU2 his3Δ1 leu2Δ0 uraΔ0*	O
YTK724B	*mud2Δ::NatNT2 ubp8Δ::KanMX4 MAT***a** *can1Δ::STE2pr-SpHIS5 lyp1Δ::STE3pr-LEU2 his3Δ1 leu2Δ0 uraΔ0*	O
YTK725A	*cus2Δ::NatNT2 ubp8Δ::KanMX4 MAT***a** *can1Δ::STE2pr-SpHIS5 lyp1Δ::STE3pr-LEU2 his3Δ1 leu2Δ0 uraΔ0*	O
YTK726A	*isy1Δ::NatNT2 ubp8Δ::KanMX4 MAT***a** *can1Δ::STE2pr-SpHIS5 lyp1Δ::STE3pr-LEU2 his3Δ1 leu2Δ0 uraΔ0*	O
YTK727A	*snu66Δ::NatNT2 ubp8Δ::KanMX4 MAT***a** *can1Δ::STE2pr-SpHIS5 lyp1Δ::STE3pr-LEU2 his3Δ1 leu2Δ0 uraΔ0*	O
YTK35	*MATα his3Δ1 leu2Δ0 ura3Δ0 met15Δ0*	A
EMy32	*bre1Δ::NatNT2 MATα his3Δ1 leu2Δ0 ura3Δ0 met15Δ0*	P
EMy442	*ubp8Δ::NatNT2 MATα his3Δ1 leu2Δ0 ura3Δ0 met15Δ0*	P
EMy489	*bre1Δ::NatNT2 nam8Δ::KanMX4 his3Δ1 leu2Δ0 ura3Δ0 met15Δ0*	G
EMy54	*bre1Δ::NatNT2 mud1Δ::KanMX4 his3Δ1 leu2Δ0 ura3Δ0 met15Δ0*	G

EMy76	*bre1Δ::NatNT2 mud2Δ::KanMX4 his3Δ1 leu2Δ0 ura3Δ0 met15Δ0*	G
EMy70	*bre1Δ::NatNT2 cus2Δ::KanMX4 his3Δ1 leu2Δ0 ura3Δ0 met15Δ0*	G
EMy81	*bre1Δ::NatNT2 lea1Δ::KanMX4 his3Δ1 leu2Δ0 ura3Δ0 met15Δ0*	G
EMy60	*bre1Δ::NatNT2 isy1Δ::KanMX4 his3Δ1 leu2Δ0 ura3Δ0 met15Δ0*	G
EMy480	*bre1Δ::NatNT2 snu66Δ::KanMX4 his3Δ1 leu2Δ0 ura3Δ0 met15Δ0*	G
EMy447	*ubp8Δ::NatNT2 lea1Δ::KanMX4 his3Δ1 leu2Δ0 ura3Δ0 met15Δ0*	G
WHY8	*3xHA-Bre1 (N-term) his3Δ1 leu2Δ0 ura3Δ0 met15Δ0 lys2Δ0*	I
N/A	*X-GFP::HIS3MX MAT***a** *his3Δ1 leu2Δ0 met15Δ02 ura3Δ0*	Q
YTK86	*NPL3 MAT***a** *his3Δ1 leu2Δ0 met15Δ0 ura3Δ0*	R
YTK87	*npl3S411A MAT***a** *his3Δ1 leu2Δ0 met15Δ0 ura3Δ0*	R

All strains are in the consortium background.

Notes:

A= Wild-type strains derived from BY4741 and BY4742 [1].

B= SGA Diploid derived from strains in [2].

C= Made by replacement of *NPL3* using techniques described by [3]. For YTK232D, this was done in YTK219, and this was followed by sporulation and selection for NAT and "magic markers." For YTK234D see [4]. Loss of *NPL3* expression was confirmed by Western blot for Npl3. YTK234D was not tested for MET/TRP markers. YTK232D was also used as a query strain in the RNA-processing E-MAP [5].

D= From KanMX4-marked MAT**a** deletion collection [6] (OpenBiosystems www.openbiosystems.com formerly Research Genetics) *X= ubp8Δ, sgf11Δ, sgf29Δ, gcn5Δ, spt3Δ, spt8Δ, ada2Δ, eaf3Δ, chd1Δ, leo1Δ, set3Δ, spp1Δ, sdc1Δ, nam8Δ, mud1Δ, mud2Δ, cus2Δ, lea1Δ, isy1Δ, snu66Δ, cdc73Δ, gbp2Δ, tho2Δ, rad18Δ, rrp6Δ, dst1Δ, ctk1Δ, set2Δ, rco1Δ, ckb2Δ, hst1Δ,* and *eaf7Δ*. PCR-validated for knockout chromosome.

E= Validated by Bill Hwang [7].

F= Wild-type ORF in YTK59 was replaced with KanMX4 using techniques from [3]. PCR-validated for knockout chromosome.

G= KanMX4-marked single mutant was mated to YTK234D (*npl3Δ::NatNT2*), EMy32 (*bre1Δ::NatNT2*), or EMy447 (*ubp8Δ::NatNT2*) and double mutant haploid was isolated using tetrad dissection. Note: unless noted, segregation of LYS, MET and TRP markers was not tested.

H= KanMX4-marked single mutant was mated to YTK234D and double mutant haploid was isolated using random sporulation. Note: segregation of LYS, MET and TRP markers was not tested.

I= Gift from Bill Hwang and Hiten Madhani.

J= YTK234D was mated to WHY334 and WHY326 and diploids were selected on YEPD + 100µg/mL hygromycin + 100µg/mL clonNAT. Following sporulation and tetrad dissection, the *htb1* gene was sequenced for the *htb1K123R* mutation and a Western blot was performed to confirm the absence of Npl3.

K= From histone H3/H4 point mutant collection [8].

L= Made by replacement of the *NPL3* ORF with KanMX4 in the H3 point mutant strains (EMy 94, 99, and 102) using techniques described in [3]. PCR validated for knockout chromosome.

M= a gift from Dale Cameron [9].

N= Starting with the cognate strains from the KanMX4-marked MAT**a** collection [6], the KanMX4 ORF was replaced with the NatNT2 ORF. The resulting strains were crossed to YTK609 and *MATα* "magic-marker" single mutants were isolated on haploid selection medium [SD medium lacking leucine and arginine but containing canavanine and s-AEC

:SD - LEU/Arg + canavanine (100µg/ml)+ S-(2-Aminoethyl)-L-cysteine hydrochloride (100µg/ml)] plus and clonNAT (100µg/mL clonNAT).

O= The *ubp8Δ::KanMX4* strain from [6] was mated to the NatNT2-marked, “magic marked” splicing factor strains. MATa double mutant haploids were isolated by tetrad dissection.

P= Wild-type ORF knocked out in YTK35 with NatNT2 using techniques from [3]. PCR-validated for knockout chromosome.

Q= From MAT**a** GFP-tagged collection [10] (Invitrogen www.invitrogen.com) X=*LUC7, RAD6, UBP8, SGF11,* and *SGF29.*

R= Unmarked NPL3 or npl3S411A alleles. From [11].

References:

1. Brachmann CB, Davies A, Cost GJ, Caputo E, Li J, et al. (1998) Designer deletion strains derived from Saccharomyces cerevisiae S288C: a useful set of strains and plasmids for PCR-mediated gene disruption and other applications. Yeast 14: 115–132. doi:10.1002/(SICI)1097-0061(19980130)14:2<115::AID-YEA204>3.0.CO;2-2.

2. Tong AHY, Boone C (2006) Synthetic genetic array analysis in Saccharomyces cerevisiae. Methods Mol Biol 313: 171–192.

3. Janke C, Magiera MM, Rathfelder N, Taxis C, Reber S, et al. (2004) A versatile toolbox for PCR-based tagging of yeast genes: new fluorescent proteins, more markers and promoter substitution cassettes. Yeast 21: 947–962. doi:10.1002/yea.1142.

4. Kress TL, Krogan NJ, Guthrie C (2008) A single SR-like protein, Npl3, promotes pre-mRNA splicing in budding yeast. Mol Cell 32: 727–734. doi:10.1016/j.molcel.2008.11.013.

5. Wilmes GM, Bergkessel M, Bandyopadhyay S, Shales M, Braberg H, et al. (2008) A genetic interaction map of RNA-processing factors reveals links between Sem1/Dss1-containing complexes and mRNA export and splicing. Mol Cell 32: 735–746. doi:10.1016/j.molcel.2008.11.012.

6. Winzeler EA, Shoemaker DD, Astromoff A, Liang H, Anderson K, et al. (1999) Functional characterization of the S. cerevisiae genome by gene deletion and parallel analysis. Science 285: 901–906.

7. Hwang WW, Venkatasubrahmanyam S, Ianculescu AG, Tong A, Boone C, et al. (2003) A conserved RING finger protein required for histone H2B monoubiquitination and cell size control. Mol Cell 11: 261–266.

8. Dai J, Hyland EM, Yuan DS, Huang H, Bader JS, et al. (2008) Probing Nucleosome Function: A Highly Versatile Library of Synthetic Histone H3 and H4 Mutants. Cell 134: 1066–1078. doi:10.1016/j.cell.2008.07.019.

9. Breslow DK, Cameron DM, Collins SR, Schuldiner M, Stewart-Ornstein J, et al. (2008) A comprehensive strategy enabling high-resolution functional analysis of the yeast genome. Nature Publishing Group 5: 711–718. doi:10.1038/nmeth.1234.

10. Huh W-K, Falvo JV, Gerke LC, Carroll AS, Howson RW, et al. (2003) Global analysis of protein localization in budding yeast. Nature 425: 686–691. doi:10.1038/nature02026.

11. Lund MK, Kress TL, Guthrie C (2008) Autoregulation of Npl3, a yeast SR protein, requires a novel downstream region and serine phosphorylation. Molecular and Cellular Biology 28: 3873–3881. doi:10.1128/MCB.02153-07.
