# Supplementary material for: The Yeast SR-Like Protein Npl3 Links Chromatin Modification to mRNA Processing
Source: PLoS Genet. 2012 Nov 29;8(11):e1003101. doi: 10.1371/journal.pgen.1003101 (PMC3510044; doi:10.1371/journal.pgen.1003101)
Supplement: Table S8 — Primers used in the qPCR assay. (PDF) [file pgen.1003101.s014.pdf]

Supplementary Table 8.
Moehle et al. "The Yeast SR-like Protein Npl3 Links Chromatin Modification to mRNA processing"
Sequences of primers used in qPCR assay (5' - 3')

LSB3 Intron F	ATACGGGTTTGGCTGGG
LSB3 Intron R	GGTTTGGTTTGACAAAACTAGC
LSB3 Total F	TGGTTCTGGTGTGATTGTCG
LSB3 Total R	CTATACCAACCATCCCACCG
MRPL44 Intron F	GCTAAACTAGGCTGAGGTAGGC
MRPL44 Intron R	GATTGAATCTGCGTGCCC
MRPL44 Total F	AGGAATTAGCCAATCATTTCG
MRPL44 Total R	GGCTTGCTTGTGAACTCTGAC
TUB1 Intron F	CATTTTGGCGTTTCATCTTTC
TUB1 Intron R	CCCAACAGGCATTACCAATC
TUB1 Total F	TCCCTTGAGCACGGTATTAAG
TUB1 Total R	TTGGAACGAACTTACCGTAGC
RPL24B Intron F	CATGAGTGTATTTCACCTTGTGG
RPL24B Intron R	GGAAAAGGAATCAACTTCAACC
RPL24B Total F	TGCTTTCCAAAAGGTTGCC
RPL24B Total R	CACAAAAATAAGTACGGATCACG
RPS7B Intron F	AGTTACTAACTTTATTTCAACGCTGC
RPS7B Intron R	GTCAATTTGGTTTGGACCTTATGG
RPS7B Total F	TGAAATTCCAAGCCAGACCAAC
RPS7B Total R	GCCCACTTTTTGGAAACCTCC
RPL31A Intron F	CCATATCCCAAGTTTGACTAAATATG
RPL31A Intron R	TCTTGAAGGAGACACCGTGC
RPL31A Total F	TAAGCAAACAGAACTCAATCAAAGG
RPL31A Total R	TGTGCAAGTTAATGGTGTATTCACG
